# (Electro)chemical N_2_ Splitting by a Molybdenum Complex with an Anionic PNP Pincer-Type Ligand

**DOI:** 10.1021/acsorginorgau.3c00056

**Published:** 2024-03-04

**Authors:** Nils Ostermann, Nils Rotthowe, A. Claudia Stückl, Inke Siewert

**Affiliations:** † Georg-August-Universität Göttingen, Institut für Anorganische Chemie, Tammannstr. 4, Göttingen 37077, Germany; ‡ Georg-August-Universität Göttingen, International Center for Advanced Studies of Energy Conversion, Tammannstr. 6, Göttingen 37077, Germany

**Keywords:** molecular electrochemistry, N_2_ splitting, molybdenum, pincer ligand, spectroelectrochemistry

## Abstract

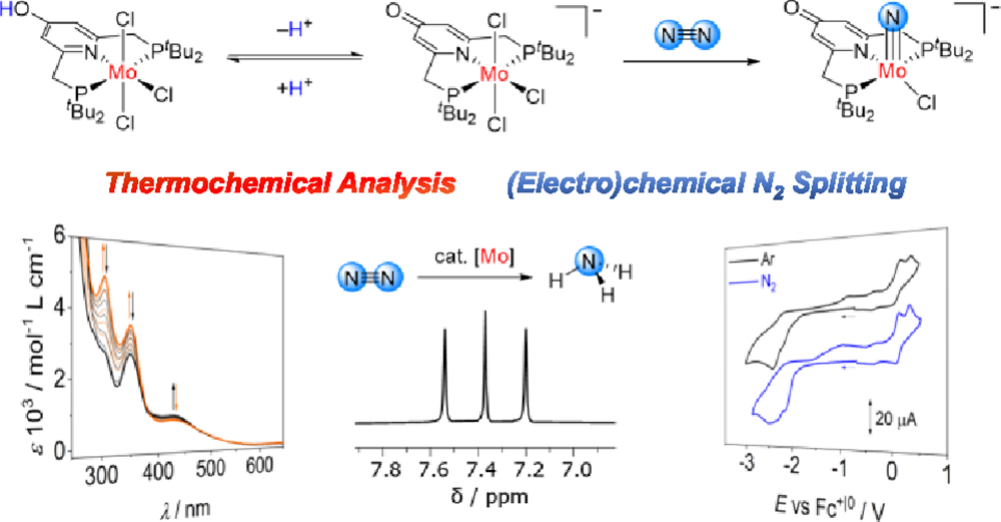

Molybdenum(III) complexes bearing pincer-type ligands are well-known catalysts for N_2_-to-NH_3_ reduction. We investigated herein the impact of an anionic PNP pincer-type ligand in a Mo(III) complex on the (electro)chemical N_2_ splitting ([*L*MoCl_3_]^−^, **1**^–^, *L*H = 2,6-bis((di-*tert*-butylphosphaneyl)methyl)-pyridin-4-one). The increased electron-donating properties of the anionic ligand should lead to a stronger degree of N_2_ activation. The catalyst is indeed active in N_2_-to-NH_3_ conversion utilizing the proton-coupled electron transfer reagent SmI_2_/ethylene glycol. The corresponding Mo(V) nitrido complex **2H** exhibits similar catalytic activity as **1H** and thus could represent a viable intermediate. The Mo(IV) nitrido complex **3**^*–*^ is also accessible by electrochemical reduction of **1**^–^ under a N_2_ atmosphere. IR- and UV/vis-SEC measurements suggest that N_2_ splitting occurs via formation of an “overreduced” but more stable [(*L*(N_2_)_2_Mo^0^)_2_μ-N_2_]^2–^ dimer. In line with this, the yield in the nitrido complex increases with lower applied potentials.

## Introduction

The reduction of dinitrogen to ammonia via the Haber-Bosch process is one of the most important processes of the last century, ensuring food production and the synthesis of nitrogen-containing organic compounds.^1^ However, the conversion of N_2_ with the chemical reductant H_2_ consumes about 1–2% of the global energy supply.^2^ Moreover, the process produces ca. 2% of annual CO_2_ emissions, which mainly originate from the formation and purification of H_2_ from steam reforming. Thus, more sustainable approaches for N_2_ fixation are highly desirable.^3^

**Scheme 1:**
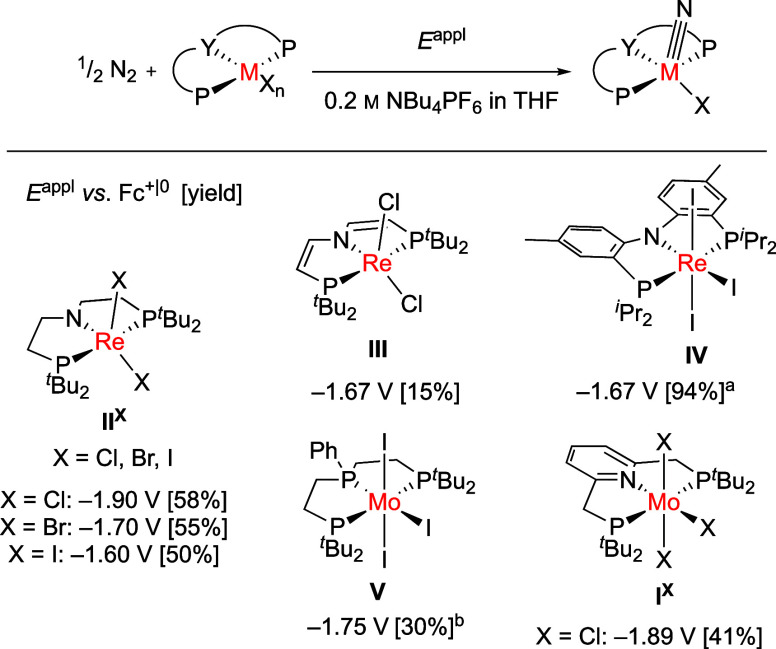
Precedents for Electrochemical N_2_ Splitting into the Corresponding Terminal Transition Metal Nitrido Complexes; Applied Potentials during Electrolysis *E*^appl^ Given vs Fc^+|0^, if Not Otherwise Noted; Yields of the Nitrido Complexes Given in Parentheses^,^ 1,2-Difluorobenzene instead of THF. Potential given vs SCE, 0.1 M NBu_4_BArF_24_ in THF.

Inspired by nature’s FeMo cofactor in the nitrogenase enzyme, the first molecular system catalyzing the N_2_-to-NH_3_ conversion was based on a well-defined molybdenum trisamidoamine complex.^4^ Profound mechanistic analysis and isolation of several intermediates led to the assumption that N_2_-to-NH_3_ formation appears by consecutive protonation and reduction steps of metal-bound N_2_.^5^ Eight years later, the group of Nishibayashi reported on a molybdenum complex with a PNP pincer-type ligand, [*L*^PNP^MoCl_3_], **I**^**Cl**^, *L*^PNP^ = 2,6-bis(^*t*^Bu_2_PCH_2_)-C_5_H_3_N) (Scheme 1), as a precursor for the corresponding Mo(0) dinitrogen complex [(*L*^PNP^(N_2_)_2_Mo)_2_μ-N_2_], that catalyzes the N_2_-to-NH_3_ reduction using cobaltocene as reductant and lutidinium as a proton source.^6^ Substitution in para position of the pyridine ligand showed that the catalytic activity is enhanced when electron-donating groups are introduced, and the authors hypothesized that this is due to a stronger degree of N_2_ activation.^7^ The TON and TOF could be dramatically increased by using the proton-coupled electron transfer (PCET) reagents SmI_2_ with alcohols or water and **I**^**Cl**^.^8^ Since the corresponding Mo(IV) nitrido complex [*L*^PNP^Mo(N)I] showed a similar catalytic performance than **I**^**I**^, the authors proposed later that the reaction with **I**^**I**^ proceeds via N_2_ splitting and subsequent reduction and protonation of the Mo nitrido complex.^9,10^ In contrast to the earlier study, complexes with electron-withdrawing substituents in para position of the pyridine unit showed enhanced activity when using the Sm-based PCET reagents.^11^

The first molecular metal catalyst beyond molybdenum was reported by the group of Peters in 2013, who introduced an iron tris(phenylphosphino)borane complex, which is active in the nitrogen reduction reaction (N_2_RR).^12^ In the following years, several more molecular N_2_-to-NH_3_ catalysts were reported based on Co, Os, Ru, Ti, V, Cr, Mo, and Re ions.^13^ These examples relied on chemical reductants, whereas electrochemical approaches for the N_2_RR remain scarce.^14^ In 1985, Pickett and Talarmin reported the first example for the electrochemical N_2_ activation forming substoichiometric amounts of ammonia using a bis(diphenylphosphinoethane) W(0) complex.^15^ In 2018, the first example of electrocatalytic N_2_RR was reported using a tris(phenylphosphino)borane Fe(I) catalyst and Cp*_2_Co^+^ as a redox mediator.^16^ The concept of combining a PCET redox mediator^17^ and a transition metal catalyst capable of accomplishing N_2_-to-NH_3_ conversion was further expanded to W, Os, Fe, and Mo complexes.^18^ Recently, the electrocatalytic N_2_RR was also accomplished with **I**^**Br**^ as a sole electrocatalyst.^19^

In Schrock’s molybdenum trisamidoamine system, catalysis appears by consecutive protonation and reduction steps of metal-bound N_2_, which contrasts with the Haber-Bosch process, where catalysis is limited by N_2_ chemisorption and splitting into surface-bound metal nitrido species.^5,20^ The first molecular model for such a metal-mediated N_2_ splitting reaction into a terminal metal nitrido complex was reported by Laplaza and Cummins using a molybdenum(III) tris-amide complex.^21^ This was followed by several more examples;^9,22^ however, examples for the electrochemically induced N_2_ splitting into terminal metal nitrido complexes remain scarce (Scheme 1).

The first example was reported by the groups of Miller, Siewert, and Schneider in 2018 using a Re(III) PNP pincer-type complex **II**^**Cl**^.^23^ [*L*^PNP2^ReCl_2_] (*L*^PNP2^ = bis(2-(di-*tert*-butylphosphaneyl)ethyl)-amide) splits dinitrogen yielding the corresponding Re(V) nitrido complex in 58% spectroscopic yield (Scheme 1). Detailed mechanistic analysis by CV (cyclic voltammetry) leads to a comprehensive, minimum model for N_2_ splitting: initial reduction of [*L*^PNP2^ReCl_2_] is followed by very fast N_2_ binding and slower chloride loss. The resulting Re(II) species is further reduced in the vicinity of the electrode due to potential inversion upon binding N_2_. Overreduced Re(I) and Re(III) form the key Re(II)–N_2_–Re(II) intermediate, which splits N_2_ to form the Re(V) nitrido complex. Changing from chlorido to bromido and iodido ligands leads to less negative potentials for N_2_-splitting along the halide series.^24^ In contrast to the chlorido system, the iodido system **II**^**I**^ reacts via a Re^II^/Re^II^-dimerization mechanism, due to the absence of the potential inversion after reduction and N_2_ binding. Utilizing the analogous unsaturated PNP ligand (**III)** increases the potential for N_2_-splitting compared to **II**^**Cl**^ with the saturated PNP ligand, but the nitrido complex was formed in a considerable lower yield (Scheme 1).^25^ To date, the highest yield of 94% could be obtained using the Re platform **IV** with the more robust diphenylamido PNP pincer-type ligand (Scheme 1).^26^

Recently, electrochemical N_2_ splitting by Mo(III) complexes supported by PPP and PNP pincer-type ligands have been reported, which form the corresponding Mo(IV) nitrido complexes in yields of 30% (**V**) and 41% (**I**^**Br**^), respectively (Scheme 1).^27,28^ Mechanistic analysis of the N_2_-splitting pathway with **I**^**Br**^ revealed that the initial reduction is followed by bromide loss and N_2_ binding. In the vicinity of the electrode, Mo(II) is further reduced forming [(*L*^PNP^BrMo^I^)_2_μ-N_2_], which splits N_2_, and overreduced [(*L*^PNP^(N_2_)_2_Mo^0^)_2_μ-N_2_], which upon a comproportionation reaction in bulk solution yields the nitrido complex.^28^

**Scheme 2:**
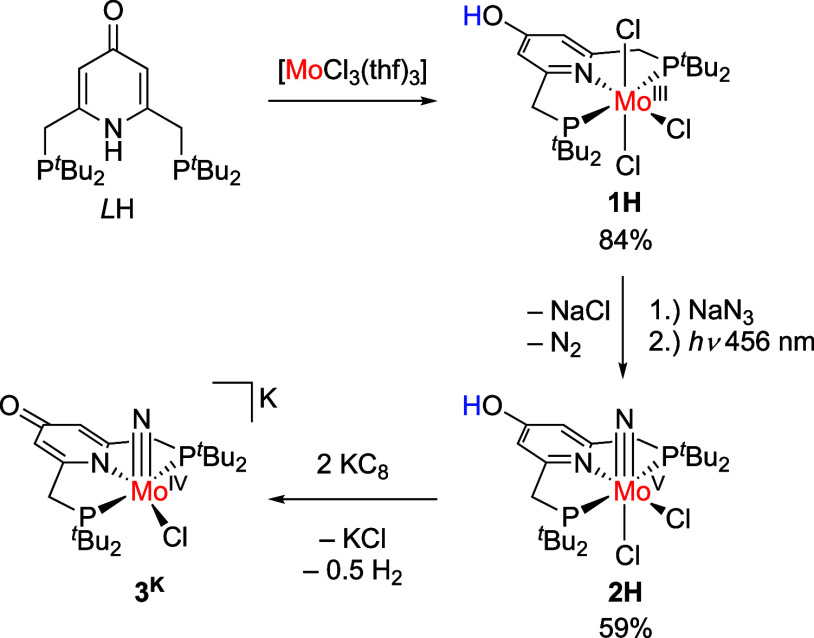
Synthesis of **1H**, **2H**, and **3**^**K**^; All Reactions in THF

Inspired by these studies, we investigated the impact of an anionic PNP-pyridone pincer-type ligand *L*^–^ on the chemical N_2_-to-NH_3_ conversion and electrochemical N_2_ splitting (Scheme 2). We expected that the increased electron-donating properties of the ligand would lead to stronger N_2_ activation in the reduced complex but also to a lower reduction potential for its formation.

## Experimental Section

If not otherwise stated, manipulations of air-sensitive reagents were carried out by means of common Schlenk-type techniques using a dry Ar or N_2_ atmosphere or performed in a N_2_- or Ar-filled MBraun glovebox. Solvents were dried using an MBraun Solvent Purification System and stored over 3 Å molecular sieves. ^15^N_2_, ^*n*^BuLi, HCl (2 M/Et_2_O), KO^*t*^Bu, NaH, DBU, ^*t*^Bu_2_PCl, NaN_3_ (all Sigma-Aldrich), and chloromethyl ethyl ether (ABCR) were used as purchased. TMEDA was dried over sodium and distilled. [MoCl_3_(thf)_3_],^29^ SmI_2_(thf)_2_,^30^ LutHOTf,^31^*L*^PNP^,^32^*L*H,^33^ and **I**^**Cl**^(6) were prepared according to literature procedures.

Electrochemical measurements (CV, DPV) were recorded with a Gamry Instruments Reference 600+ or Reference 600 in dry THF under a N_2_ or Ar atmosphere. A common three-electrode setup was used with a glassy carbon working electrode (GC: CH Instruments, ALS Japan; *A* = 7.1 mm^2^), a platinum wire as a counter electrode, and a silver wire in electrolyte solution in a fritted sample holder as a pseudo reference electrode. The GC electrode was polished on an alumina polishing pad with 0.05 μm polishing alumina (both ALS Japan) and Milli-Q water in figure-eight motion. The platinum and silver wires were washed with Milli-Q water. ^*n*^Bu_4_NPF_6_ was dried at 100 °C *in vacuo* and used as the conducting salt, *I* = 0.2 M. All data were collected at RT and referenced internally versus the Fc^+|0^ or Fc*^+|0^ redox couple (conversion of Fc*^+|0^ to Fc^+|0^ –0.43 V). i*R* compensation was performed by the positive feedback method, which is implemented in the PHE200 software of Gamry. All CV data are plotted in IUPAC convention.

### [(*L*H)MoCl_3_] (**1H**)

A suspension of [MoCl_3_(thf)_3_] (112 mg, 0.27 mmol, 1.00 equiv) and *L*H (116 mg, 0.28 mmol, 1.05 equiv) was heated in THF (10 mL) at 45 °C for 18 h under an Ar atmosphere. The solution was filtered, and the solvent was removed *in vacuo*. Recrystallization from CH_2_Cl_2_/hexane afforded **1H** (139 mg, 0.23 mmol, 84%) as an orange/brown powder. Crystals suitable for X-ray diffraction were obtained by vapor diffusion of pentane into a solution of **1H** in THF at −30 °C. HR-MS (ESI): [C_23_H_43_Cl_2_MoNOP_2_]^+^ ([M–Cl]^+^): calcd. *m*/*z* = 579.1239, found *m*/*z* = 579.1231. [C_23_H_43_Cl_3_MoNNaOP_2_]^+^ ([M+Na]^+^): calcd. *m*/*z* = 637.0819, found *m*/*z* = 637.0813. μ_eff_ = 3.8 μ_B_. IR (KBr): ν̃ (cm^–1^) = 3250 (br), 2949 (m), 2900 (m), 2870 (m), 1614 (s), 1593 (m), 1475 (s), 1394 (w), 1369 (m), 1342 (m), 1261 (w), 1219 (w), 1178 (m), 1146 (w), 1105 (w), 1024 (s), 974 (w), 937 (w), 868 (m), 812 (m). El. anal. for C_23_H_43_Cl_3_MoNOP_2_: C, 45.00; H, 7.06; N, 2.28. Found: C, 44.70; H, 6.60; N, 2.15.

### [(*L*H)MoCl_2_N] (**2H**)

NaN_3_ (26.5 mg, 0.41 mmol, 2.50 equiv) was added to a solution of **1H** (100 mg, 0.16 mmol, 1.00 equiv) in THF (5 mL) under a N_2_ atmosphere. The solution was stirred overnight, filtered over Celite, and dried *in vacuo*. The residue was recrystallized from CH_2_Cl_2_/hexane at −30 °C and washed with Et_2_O (3 × 2 mL). The resulting solid was dissolved in THF (3 mL, 25 mL Schlenk tube, borosilicate 3.3 glass), stirred, and irradiated with a 456 nm LED overnight (Kessil PR160L-456 nm, distance 5 cm). The resulting solution was filtered and recrystallized from THF/hexane at −30 °C to obtain **2H** (57.2 mg, 0.97 mmol, 59%) as a brown/yellow powder. HR-MS (ESI): [C_23_H_43_ClMoN_2_OP_2_]^+^ ([M–Cl]^+^): calcd. *m*/*z* = 558.1586, found *m*/*z* = 558.1589. IR (KBr): ν̃ (cm^–1^) = 3255 (br), 2949 (m), 2899 (m), 2866 (m), 1591 (s), 1472 (s), 1391 (w), 1367 (m), 1342 (m), 1261 (w), 1221 (w), 1175 (m), 1138 (w), 1030 (m), 935 (w), 866 (w), 810 (w). El. anal. calcd. for C_23_H_43_Cl_2_MoN_2_OP_2_: C, 46.63; H, 7.32; N, 4.73. Found: C, 46.0; H, 7.23; N, 4.73.

### General Procedure for Catalytic N_2_ Reduction

Dry THF (6 mL) was added to a Schlenk tube filled with the catalyst (2.00 μmol, 1.00 equiv) and SmI_2_(thf)_2_ (197 mg, 360 μmol, 180 equiv) in a N_2_ glovebox. Ethylene glycol (22.4 mg, 360 μmol, 180 equiv) was added in one portion, and the mixture was further stirred for 18 h at RT resulting in a color change from deep blue to yellow. The mixture was frozen to −196 °C, and an excess of KO^*t*^Bu (∼100 mg) in MeOH (5 mL) was added. The mixture was allowed to warm to room temperature and stirred for 15 min. The volatiles were vacuum transferred into a liquid N_2_-cooled Schlenk flask containing HCl (2 M in Et_2_O, 3 mL). The solution was stirred for a further 15 min at RT, the solvents were removed *in vacuo*, and the residue was dissolved in DMSO-*d*_6_ with trimethoxybenzene as an internal standard. The amount of ammonium was determined by integration versus the internal standard.

### Electrolysis Experiments

Controlled potential electrolysis (CPE) experiments were performed in a custom-made H-type cell with a P3 glass frit to separate working and counter chambers under a N_2_ atmosphere. The working chamber was equipped with a 7 mm-diameter glassy carbon rod (ALS Japan) and a silver wire in electrolyte solution in a fritted sample holder as a pseudo reference electrode. The counter chamber was equipped with a Zn rod as a sacrificial electrode. Both chambers were filled with 2.5 mL of electrolyte solution (0.2 M ^*n*^Bu_4_NPF_6_ in THF), and a stirring bar was added. **1H** (12.3 mg, 20 μmol) and DBU (3.00 μL, 3.04 mg, 20 μmol) were added to the working chamber. A fixed potential was applied, and the solution was electrolyzed until the desired amount of charge was passed. An aliquot of the solution in the working chamber was taken, and a drop of THF-*d*_8_ was added. The mixture was analyzed by ^31^P NMR spectroscopy, and **3**^**–**^ was quantified by integration vs the PF_6_^–^ anion.

## Results and Discussion

### Synthesis and Characterization of the Complexes

The synthesis of the PNP pincer-type ligand *L*H was previously reported.^33^ The reaction of [MoCl_3_(thf)_3_] and *L*H at 45 °C in THF yielded paramagnetic molybdenum complex **1H** in 84% yield after purification (Scheme 2). The complex was isolated as an orange-brown solid. IR spectroscopy showed a band at a frequency of 3250 cm^–1^ indicative of an O–H stretching vibration, and positive ESI-MS showed the presence of the [M–Cl]^+^ and [M+Na]^+^ ions.

**Figure 1:**
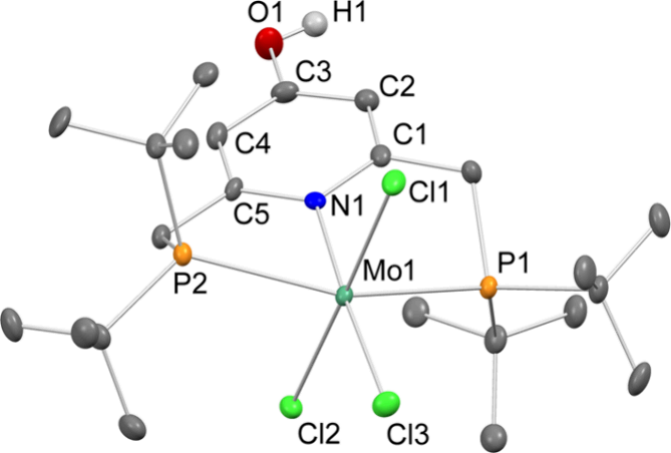
Molecular structure of **1H**; the hydrogen atom was placed in a calculated position. Most hydrogen atoms and all solvent molecules were omitted for clarity. Thermal ellipsoids were set at the 50% probability level.

The magnetic moment μ_eff_ of **1H** derived by the Evans method is 3.8 μ_B_, which is characteristic for an *S* = 3/2 ground state (*S* = 3/2: μ_SO_ = 3.87 μ_B_). The EPR spectrum of a THF solution of **1H** recorded at 293 K shows hyperfine coupling to two phosphorus and one molybdenum atom (*g*_iso_ = 2.0093, *A*(2 × ^31^P) = 67 MHz, *A*(1 × ^95/97^Mo) = 76 MHz; Figure S16). Single X-ray crystals of the compound were obtained by vapor diffusion of pentane into a solution of **1H** in THF. The results of the refinement can be found in Figure 1, selected bond lengths are listed in Table 1, and further details are given in the SI.

**Table 1:** Selected Bond Lengths (Å) for **1H**, with Estimated Standard Deviations in Parentheses

bond	distance	bond	distance
Mo–Cl1	2.4103(12)	Mo–N1	2.189(4)
Mo–Cl2	2.4108(11)	Mo–P1	2.6121(12)
Mo–Cl3	2.4515(12)	Mo–P2	2.6235(11)
C3–O1	1.352(5)	C1–C2	1.373(6)
C4–C5	1.384(6)		

The molybdenum ion exhibits a distorted octahedral coordination sphere. The P–Mo–P angle is 153.32(4)°, and the N–Mo–Cl3 angle is 175.92(11)°. The Mo–N distance is 2.189(4) Å, and the Mo–P distances are 2.6235(11) and 2.6121(12) Å, which are very similar to the Mo–X distances in the corresponding Mo(III) complexes with 2,6-bis((di-*tert*-butylphosphanyl)methyl) pyridine ligands bearing different substituents in the para position of the ligand backbone.^6,7^ The elongated C–O bond of 1.352(5) Å as well as the C1–C2 and C4–C5 distances of 1.373(6) Å and 1.384(6) Å, respectively, support protonation of the ligand.^33,34^ This is in line with elemental analyses, which confirm the purity of **1H**.

**Figure 2:**
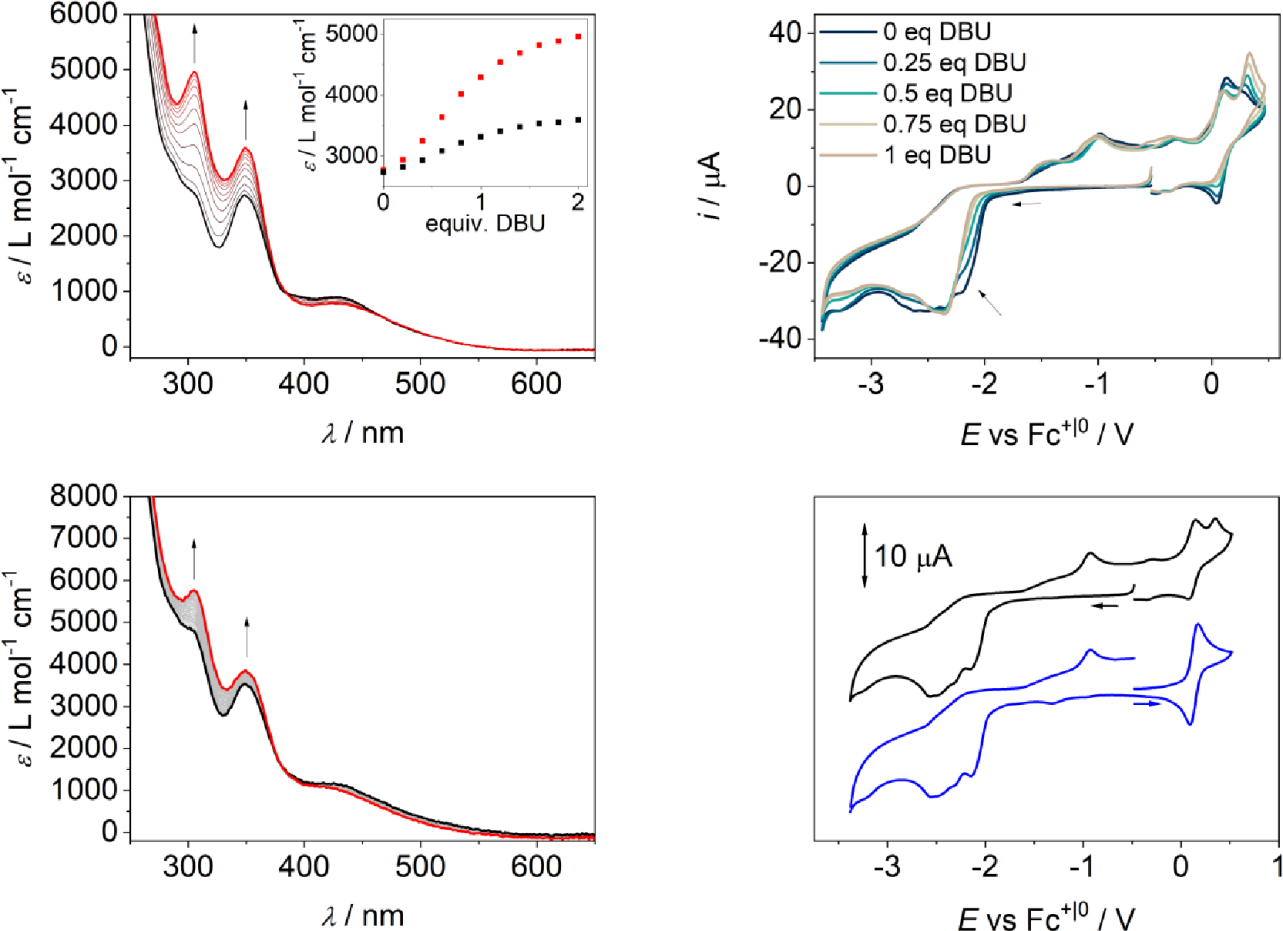
Top left: UV/vis titration of **1H** with increasing amounts of DBU (black: 0 equiv, red: 2 equiv), THF, *c*_**1H**_ = 0.15 mM; the inset shows the extinction coefficient at 305 nm (red) and 349 nm (black) vs the equivalents of DBU. Top right: CV data of **1H** with varying amounts of DBU, *c*_**1H**_ = 1 mM, ν = 0.1 V s^–1^, *I* = 0.2 M ^*n*^Bu_4_NPF_6_, THF, N_2_. Bottom left: UV/vis-SEC spectra recorded during reduction of **1H** at an applied potential of −2.4 V vs Fc^+|0^ (black: beginning, red: end), THF, *c*_**1H**_ = 0.15 mM, *I* = 0.2 M ^*n*^Bu_4_NPF_6_. Bottom right: CV data of **1H** under N_2_, *c*_**1H**_ = 0.5 mM, ν = 0.1 V s^–1^, *I* = 0.2 M ^*n*^Bu_4_NPF_6_, THF.

Since the spectroscopic features of **1H** pointed to the formation of the neutral Mo(III) complex, we investigated the deprotonation of the ligand via UV/vis spectroscopy. Upon gradual addition of DBU, the π–π* band at 349 nm increases and the shoulder at 305 nm gets more prominent (DBU = 1,8-diazabicyclo[5.4.0]undec-7-ene, p*K*_a_ = 16.9,^35^Figure 2). The d–d band at 430 nm decreases very little. Fitting of the data at five representative wavelengths led to a p*K*_a_ of 15.9 ± 0.2 for **1H** in THF (Figure S1). Back-titration with lutidinium reproduces the initial spectrum of **1H** with a spectroscopic yield of >90%, revealing reversible protonation and deprotonation (Figure S1).

The CV and DPV (differential pulse voltammetry) data of **1H** show a reversible Mo^III^/Mo^IV^ oxidation at *E*^1/2^ = 0.13 V (Figure 2 and Figure S4; if not otherwise noted, a glassy carbon (GC) electrode was used and the data are given vs the Fc^+|0^ reduction potential). The Mo^III^/Mo^IV^ oxidation appears only at a slightly lower potential than in parent complex **I**^**Cl**^ (*cf*. *E*^1/2^ = 0.20 V, Figure S7) indicating that switching from hydroxypyridine to pyridine has very little effect on the electronic structure at the Mo ion. Scanning reductively, **1H** shows an irreversible reduction at *E*_p,c,2_ = −2.41 V with a shoulder at *E*_p,c,1_ = −2.19 V and three associated irreversible reoxidations at *E*_p,a,1_ = −0.85 V, *E*_p,a,2_ = −0.31 V, and *E*_p,a,4_ = 0.35 V under Ar (ν = 0.1 V s^–1^, Figure S3). Both reduction events remain irreversible upon increasing the scan rate from 0.05 to 1 V s^–1^ (Figure S2). The irreversibility of all reduction events irrespective of the scan rate is in sharp contrast to **I**^**Cl**^ and **I**^**Br**^, which showed an increased reversibility of the first reduction upon increasing the scan rate (Figure S7 and ref (28)). Initial reduction of **I**^**Br**^ was proposed to be followed by rather slow bromide loss.^28^ This points to a different reactivity after the reduction of **1H**.

Based on previous reports with such hydroxypyridine ligands, we envisioned reductive O–H bond cleavage as a follow-up reaction as observed, e.g., by Fujita and co-workers in Re and Ru complexes bearing the [2,2′-bipyridine]-4,4′-diol ligand.^36^ Upon reduction, homolytic O–H bond cleavages occur, forming the dianionic bipyridone ligand and H_2_. A similar scenario seemed plausible also for **1H**, and indeed, a UV/vis-spectroelectrochemical (SEC) experiment under reductive conditions revealed similar changes in the UV/vis spectra as observed during deprotonation of **1H** (Figure 2 and Figure S12). Analysis of the gas phase by gas chromatography after a CPE experiment at an applied potential of −2.1 V passing 1 charge equivalent revealed the formation of H_2_ in yields of 55% (Figure S38). CV studies of **1H** with increasing amounts of DBU showed the disappearance of the first reduction wave, which was therefore assigned to reductive O–H bond cleavage forming H_2_ and **1**^**–**^ (Figure 2 and Figure S6). Further reduction of deprotonated complex **1**^**–**^ overlaps with the initial reduction of **1H** and could explain the low yield in H_2_.

The CV and DPV data of *in situ* formed **1**^–^ show a Mo^III^/Mo^IV^ oxidation at *E*^1/2^ = 0.07 V (Figure 2 and Figures S4 and S6). The potential shift compared to the oxidation of **1H** is small indicating a weak thermodynamic coupling between the proton and the metal site, similar to that observed for the respective Ni complex.^33^ The CV data of **1**^–^ show a second irreversible oxidation at a peak potential of 0.36 V, which likely belongs to the oxidation of the deprotonated ligand, because it becomes more prominent in **1H** with increasing amounts of DBU and it is also present in the CV data of **1H**, when the CV is scanned reductively at first, but not present, when scanned anodically at first (Figure 2 and Figures S5 and S6).

Having established the p*K*_a_ of **1H** and the oxidation potential of **1**^–^, we estimated the BDFE of the O–H bond in **1H** from the Bordwell equation using the recently reported value of *c*_G_ in THF with 59.9 kcal mol^–1^.^37^
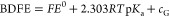


It sums up to 83 kcal mol^–1^, which is similar to the BDFE of the O–H bond in phenol.^37^

Treatment of **1H** with a slight excess of NaN_3_ leads to the azido complex, which upon irradiation with a blue LED at a wavelength of 456 nm in THF provides the Mo(V) nitrido complex **2H** in 59% yield after recrystallization from THF/hexane (Scheme 2). High-resolution ESI mass spectrometry showed the formation of [(*L*H)Mo(N)Cl]^+^. The EPR spectrum of a THF solution of **2H** at 279 K showed one signal at *g*_iso_ = 1.9849, and simulation as an *S* = 1/2 system revealed hyperfine coupling to two different N atoms and the phosphorus atoms substantiating formation of the desired Mo(V) nitrido complex **2H** (*A*(2 × ^31^P) = 40.6 MHz; *A*(2 × ^1^H) = 4.2 MHz; *A*(1 × ^14^N) = 9.8 MHz; *A*(1 × ^14^N) = 4.5 MHz, Figure S17). The purity of **2H** was confirmed by an elemental analysis. **2H** exhibits two irreversible reductions at peak potentials of −2.38 and −2.59 V and four oxidation processes at peak potentials of −0.91, −0.28, 0.15, and 0.39 V (ν = 0.1 V s^–1^, Figure S8). The oxidation at −0.91 V was assigned to the Mo(IV/V) redox couple in analogy to related Mo nitrido complexes.^9,27,38^ Reduction of **2H** with an exc. of KC_8_ or deprotonation of **2H** with 1 equiv of DBU and reduction with an exc. of KC_8_ led to the corresponding Mo(IV) nitrido complex **3K**, which shows a characteristic signal in the ^31^P NMR spectrum at 85.2 ppm (Scheme 2 and Figure S19).^39^ The CV data of **3**^**–**^ exhibit no characteristic reduction feature up to −2.5 V and oxidation processes at peak potentials of −1.13, −0.36, and 0.65 V (ν = 0.1 V s^–1^, Figure S 9)_._

### Reactivity towards N_2_ Using Chemical Reductants

Next, we focused on the catalytic reactivity of **1H** in the dinitrogen reduction reaction forming ammonia. Therefore, we adapted the previously reported protocol for the N_2_-to-NH_3_ formation catalyzed by **I**^**I**^.^8^**1H** was treated with 180 equiv SmI_2_(thf)_2_ and 180 equiv ethylene glycol in THF under a 1 atm nitrogen atmosphere for 18 h (Table 2). This led to formation of 9.4 ± 0.6 equiv of ammonia per Mo, confirming that **1H** is indeed an active catalyst in the N_2_-to-NH_3_ formation, albeit with lower TON than **I**^**I**^. Ammonia was quantified as the ammonium salt after vacuum transfer and acidic workup via NMR spectroscopy with the internal standard 1,3,5-trimethoxybenzene. Repeating the experiment under a ^15^N_2_ atmosphere led to the expected ^1^H signal of ^15^NH_4_^+^, confirming that N_2_ is the N source in ammonium (Table 2 and Figures S20 and S21). A blank experiment in the absence of the catalyst did not lead to detectable amounts of NH_3_.

**Table 2:**
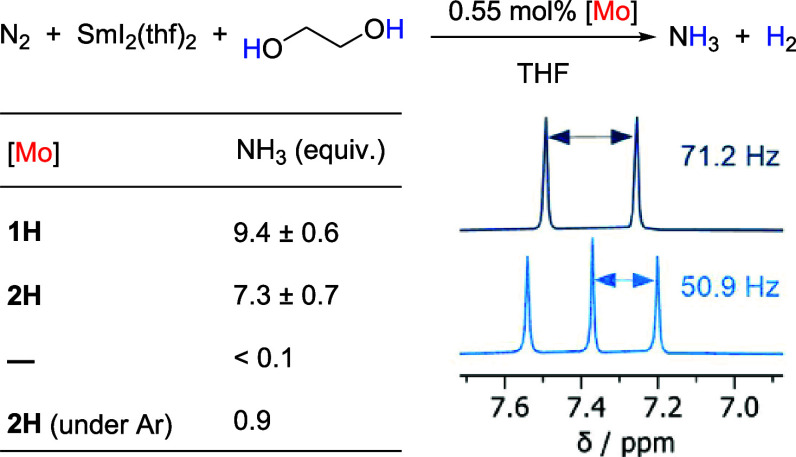
Catalytic N_2_-to-NH_3_ Conversion^a^

a Inset: ^1^H NMR spectra in DMSO-*d*_6_ of the ammonium cation generated via N_2_ reduction under ^14^N_2_ (bottom) and ^15^N_2_ (top) catalyzed by **1H**.

To get further insights into the N_2_ reduction pathway, the catalytic activity of the corresponding molybdenum(V) nitrido complex **2H** was investigated next. Reaction of **2H** under the same conditions showed catalytic conversion of dinitrogen into ammonia with a TON of 7.3 ± 0.7, indicating that the nitrido complex could be a plausible intermediate during catalysis with **1H**, although the yield was slightly lower (Table 2). Control experiments under an argon atmosphere with **2H** showed the formation of 0.9 equiv of ammonia per catalyst, which originates from the nitrido nitrogen atom. Catalysis under isotopically labeled ^15^N_2_ with **2H** revealed catalytic conversion into ^15^NH_3_ and 1 equiv of ^14^NH_3_ (Figure S23).

### Reactivity towards N_2_ under Electrochemical Conditions

To probe for electrochemical N_2_ splitting, CV data under a N_2_ atmosphere were recorded. Under a N_2_ atmosphere, **1H** shows four irreversible reductions at *E*_p,c,1_ = −2.15 V, a shoulder at *E*_p,c,2_ = −2.32 V, and a third and fourth event at *E*_p,c,3_ = −2.46 V and *E*_p,c,4_ = −2.57 V, respectively (ν = 0.1 V s^–1^, Figure 2). The second reduction process shifts largely with increasing scan rates and merges with the third one at 1 V s^–1^ (Figure S5). When the potential is switched beyond the third reduction process, two new oxidation processes appear at *E*_p,a,1_ = −0.93 V and at *E*_p,a,2_ = 0.35 V, as well as the Mo(III/IV) oxidation at *E*^1/2^ = 0.13 V. The oxidation process at −0.93 V is not present when the CV data of **1H** are collected under Ar, and the CV data of **2H** exhibit an oxidation process at a similar potential (Figures S8 and S9), which could indicate *in situ* formation of the nitrido complex. The latter new oxidation process *E*_p,a,2_ belongs to the oxidation of the deprotonated ligand (*vide supra*). As under Ar, initial reduction is associated with the cleavage of the O–H bond and the formation of **1**^–^ as confirmed by titration experiments with DBU. The reduction vanishes with increasing amounts of DBU (Figure S6). The first reduction of **1H** under N_2_ appears at a potential −0.2 V lower than the potential of the first reduction of **I**^**Cl**^, whereas further reductions of **1H** are easier to accomplish than in **I**^**Cl**^ (*cf*. **I**^**Cl**^: *E*^1/2^ = −1.94 V, *E*_p,c,1_ = −2.48 V, *E*_p,c,2_ = −2.71 V, *E*_p,c,3_ = −3.03 V, ν = 0.1 V s^–1^, Figure S7).

Since the CV data under a N_2_ atmosphere revealed distinct differences compared to those under Ar, electrochemical dinitrogen splitting was investigated. As initial reduction is associated with H_2_ formation, we utilized **1**^–^ in the electrochemical N_2_ splitting experiments, which was prepared *in situ* by adding one equivalent of DBU. **1**^–^ was electrolyzed at −2.69 V vs Fc^+|0^ until four electrons were injected. Notably, [DBUH]^+^ is reduced at the applied potential of the electrolysis; thus, only three electrons are consumed by **1**^–^ (Figure S10). Independent CPE experiments of [DBUH]Cl at an applied potential of −2.6 V confirmed its one-electron reduction and H_2_ evolution in yields of 75% (Figures S10 and S11). ^31^P NMR spectroscopy revealed the formation of a singlet at a chemical shift of 84.4 ppm corresponding to the desired Mo(IV) nitrido complex **3**^**–**^ in only 7% yield. The yield has been quantified via integration vs the PF_6_^–^ signal (Table 3, entry 1, and Figure S25). Since reductive N_2_ splitting by **1**^–^, leading to the Mo(IV) nitrido complex, requires only 2 e^–^, we investigated the yield in dependence of the injected charge equivalents per [Mo], i.e., 2, 3, and 4 e^–^. After the injection of two electrons, the formation of the Mo(IV) nitrido complex was not observed. This is in line with the electronic structure requirements for N_2_ splitting in a pseudo *D*_4h_ symmetric complex (*vide infra*). After injection of three electrons, it was formed in higher yields of 12%. Injection of 4 e^–^ leads to lower yields, indicating that overreduction leads to decomposition or formation of paramagnetic species (Table 3, entries 2, 3, and 5, and Figure S29). CV analysis of **2H** showed that the Mo nitrido complex is reduced at the applied potential of the electrolysis, and thus, it could be further reduced *in situ* forming paramagnetic species (Figure S8). To probe if ^31^P NMR spectroscopy is a suitable method to quantify nitrido complex formation, it was also quantified via release as ammonium and subsequent ^1^H NMR spectroscopy (Figure S30). Ammonium formation was observed after workup with a spectroscopic yield of 14%, confirming the reliability of quantification by ^31^P NMR spectroscopy (Table 3, entry 4). Electrochemical reduction of **1H** under a ^15^N_2_ atmosphere and subsequent acidic workup confirmed that N_2_ is the N source in ammonium (Figure S31).

**Table 3:** Yields of the Mo(IV) Nitrido Complex after Electrochemical Reduction of **1H** with 1 Equiv DBU in THF under N_2_

no.	*E*^appl^ vs Fc^+|0^	number of e^–^	quantified by	yield (%)
1	–2.69 V	4	^31^P NMR	7
2	–2.82 V	2	^31^P NMR	0
3	–2.82 V	3	^31^P NMR	12
4	–2.93 V	3	NH_3_ release	14
5	–2.82 V	4	^31^P NMR	4
6	–3.08 V	5	^31^P NMR	18
7	–3.15 V	3	^31^P NMR	31^a^

a Boron-doped diamond electrode instead of GC.

**Figure 3:**
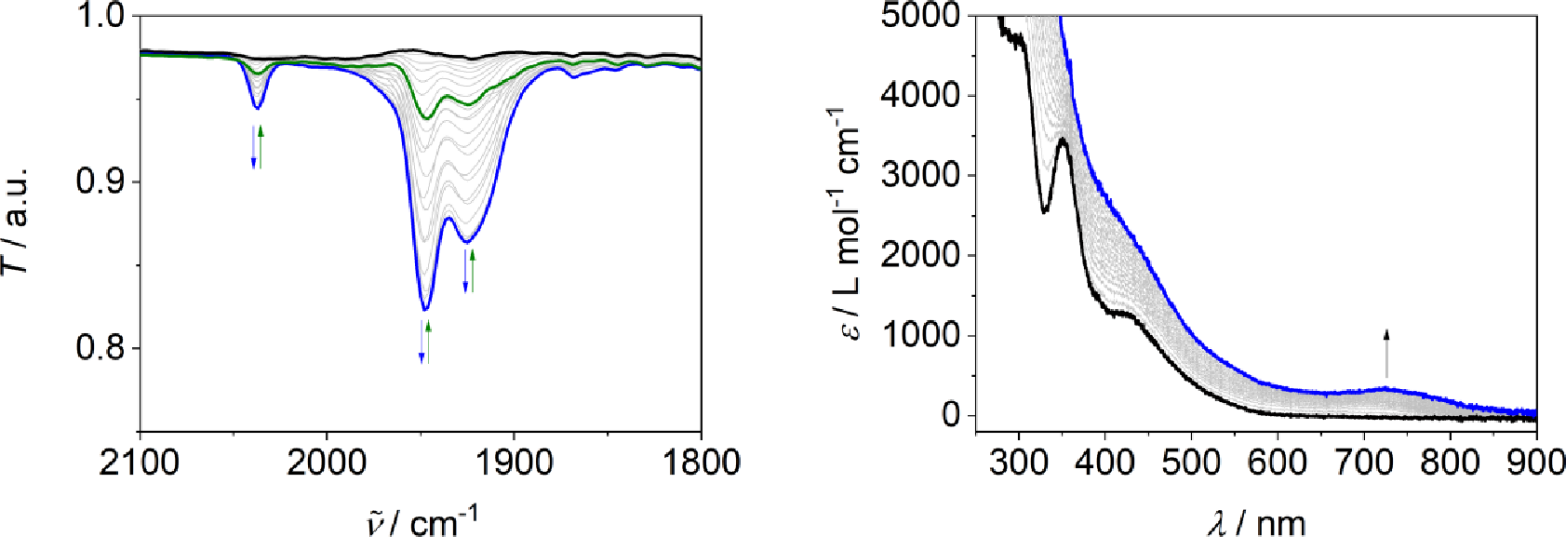
Left: IR spectra recorded during reductive IR-SEC of **1H**, *v* = 0.0025 V s^–1^, *c*_**1H**_ = 10 mM; black: initial, blue: middle, green: last spectrum. Right: UV/vis spectra recorded during UV/vis-SEC of **1H** at an applied potential of −2.7 V vs Fc^+|0^, *c*_**1H**_ = 0.15 mM; black: initial spectrum; blue: last spectrum. Both THF, *I* = 0.2 M ^*n*^Bu_4_NPF_6_, N_2_.

To obtain further insights into the mechanism, IR-spectroelectrochemical investigation of **1H** under reductive conditions under a N_2_ atmosphere in an OTTLE cell using a gold grid electrode were conducted.^40^ The IR data showed the appearance of three bands at 2037, 1947, and 1925 cm^–1^ upon reduction (Figure 3). These bands are assigned to the vibrations of terminal N≡N triple bonds in the Mo(0) monomer [LMo(N_2_)_3_]^−^ (ν̃_NN_ = 1947 and 2037 cm^–1^) and Mo(0) dimer [(L(N_2_)_2_Mo)_2_μ-N_2_]^2–^ (ν̃_NN_ = 1925 cm^–1^), because similar bands appeared during electrochemical and chemical reduction of **I**^**Br**^ and have been assigned to an equilibrium of the monomer [L^PNP^Mo(N_2_)_3_] (ν̃_NN_ = 1963 and 2046 cm^–1^) and the dimer [(L^PNP^(N_2_)_2_Mo)_2_μ-N_2_] (ν̃_NN_ = 1944 cm^–1^).^28^ These bands disappear during a prolonged electrolysis time (Figure S14). The N_2_ stretching vibrations of the intermediates during N_2_ splitting with **1**^–^ are shifted to lower energy compared to those utilizing **I^Br^**, supporting the assumption that the anionic ligand led to a higher N_2_ activation degree. The vibrations were unambiguously assigned to N_2_ vibrations by using isotope-labeled ^15^N_2_. The stretching vibrations are red-shifted and appear at 1967, 1875, and 1853 cm^–1^ (Figure S15). Assuming a similar force constant, the vibrations are expected at 1968, 1881, and 1859 cm^–1^ as calculated from the change of the reduced mass in ^14/15^N_2_, which fits well with the experimental data.

The formation of a dinitrogen bridged Mo(0) dimer as an intermediate during nitrido formation was further substantiated by UV/vis-SEC measurements. During electrolysis, a new band at 723 nm appeared, which is characteristic for the dimer [(*L*(N_2_)_2_Mo)_2_μ-N_2_]^2–^ comparing it with the parent [(*L*^PNP^(N_2_)_2_Mo)_2_μ-N_2_] complex, which has a characteristic band at 688 nm (Figure 3 and Figure S13).^28^

**Scheme 3:**
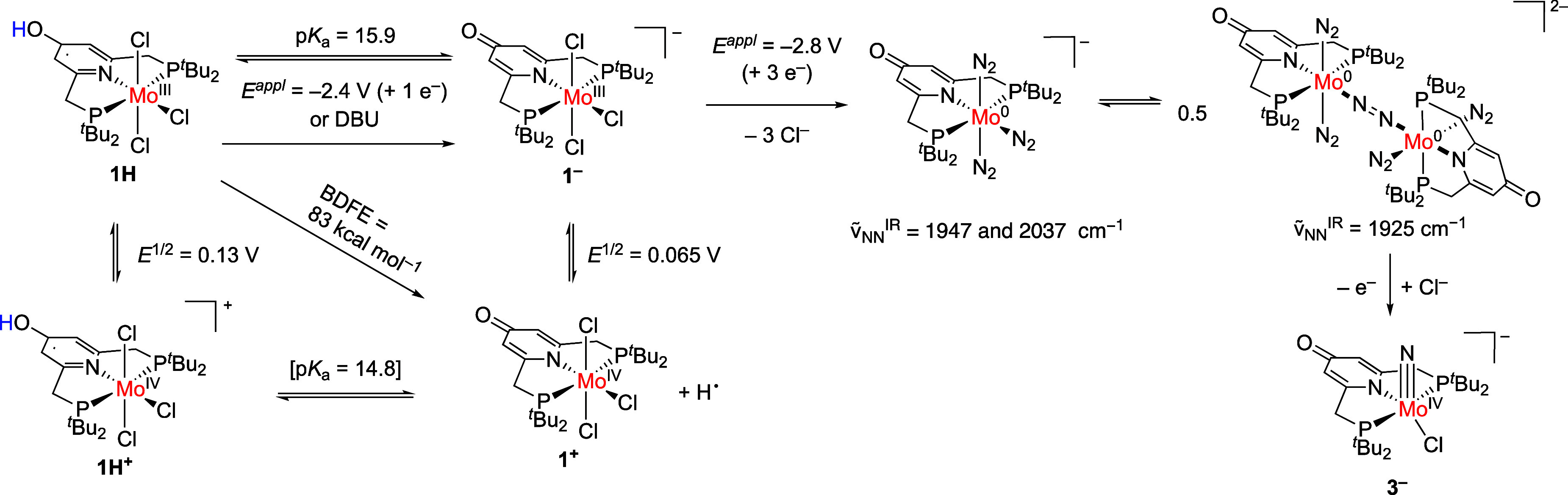
Thermochemical Data of **1H** and Proposed Mechanism for Electrochemical N_2_ Splitting Forming the Mo Nitrido Complex **3**^–^

Considering the electronic structure requirements for N_2_ splitting by dimeric [(M)_2_μ-N_2_] species in a pseudo *D*_4h_ symmetry, Mo(I) should be the active species as it exhibits the π^10^ configuration, which leads to the maximum weakening of the N_2_ bond and the electronic requirements for forming the metal nitrido species.^41^ In line with these considerations, electrochemical oxidation of *trans*-(depe)_2_Mo^0^(N_2_)_2_ induces N_2_ splitting forming the nitrido complex.^42^ However, IR-SEC experiments of **1H** under N_2_ point to the *in situ* formation of Mo(0) species, indicating an overreduction of the complex. Such electrochemical overreduction in the vicinity of the electrode surface has been proposed previously in related Mo and Re complexes with PNP pincer-type ligands on the path to nitrido complex formation.^23,28^ This was rationalized by the potential inversion of the reduction potentials after halide loss and N_2_ binding. Overreduced Mo(0) or Re(I) reacts with the starting material forming the reactive intermediate, *viz*., the Re(II)- or Mo(I)-bridged dimer, respectively. A similar scenario seems also plausible here, as **1**^–^ shows very similar potentials for successive reduction steps under a N_2_ atmosphere.^43^ Oxidation of the Mo(0) dimer by homogeneous electron transfer from, e.g., **1**^–^ induces N_2_ splitting forming the Mo(IV) nitrido complex (Scheme 3). Such overreduction forming a more stable Mo(0) intermediate as a viable route was further examined by potential dependent formation of the nitrido complex. At an applied potential of −2.69 V, molybdenum nitrido formation was observed in 7% yield (Table 3, entry 1). With lower applied potentials, the yield increases to 18% at −3.08 V and 31% at −3.15 V (Table 3, entries 6 and 7), which is in line with the assumption that the overreduction is crucial for N_2_ splitting formation.

## Conclusions

The molybdenum(III) complex **1**^–^ with an anionic PNP pyridone pincer-type ligand has been investigated in N_2_-to-NH_3_ conversion and electrochemical N_2_ splitting. The complex catalyzes the ammonia formation with SmI_2_/ethylene glycol as a PCET reagent with a TON of 9.4. The reaction could proceed via the Mo nitrido complex, from which ammonia is released by successive protonation and reduction steps, as we observe very similar TON in the experiments using **1H** and the Mo(V) nitrido complex **2H**. Electrochemical reduction of **1**^–^ in the presence of N_2_ leads to the formation of the Mo(IV) nitrido complex **3^–^**. IR- and UV/vis-SEC experiments indicate that the reaction proceeds via an overreduced Mo(0) complex. In line with this, the yield in **3**^**–**^ increases with a lower applied potential. The anionic nature of the ligand shifts the potential for the initial reduction of **1**^–^ to more negative potential compared to **I**^**Cl**^; however, the degree of N_2_ activation seems to be slightly higher than in the parent complex due to the more electron-donating properties of the ligand as deduced from the N≡N stretching vibrations in the molybdenum–dinitrogen complexes.

## Data Availability

The data underlying this study are available in the published article and its Supporting Information.

## Supporting Information Available

Instrumentation; syntheses; UV/vis titration of **1H**; electroanalytical data; UV/vis spectroelectrochemistry of **1H**; IR spectroelectrochemistry of **1H**; EPR spectra; NMR spectra; data of the electrochemical N_2_ splitting with **1**^**–**^; IR spectra; mass spectra; quantification of H_2_; and X-ray crystallography (PDF)
