# Supplementary material for: (Electro)chemical N2 Splitting by a Molybdenum Complex with an Anionic PNP Pincer-Type Ligand
Source: ACS Org Inorg Au. 2024 Mar 4;4(3):329–37. doi: 10.1021/acsorginorgau.3c00056 (PMC11157508; doi:10.1021/acsorginorgau.3c00056)
Supplement: Supplementary file 1 — gg3c00056_si_001.pdf [file gg3c00056_si_001.pdf]

Supporting Information

(Electro)chemical N_2 Splitting by a Molybdenum Complex with an Anionic PNP Pincer-Type Ligand

Nils Ostermann,[1] Nils Rotthowe,[1] A. Claudia Stückl,[1] Inke Siewert[1,2*]

1: Georg-August-Universität Göttingen, Institut für Anorganische Chemie, Tammannstr. 4, 37077 Göttingen, Germany

2: Georg-August-Universität Göttingen, International Center for Advanced Studies of Energy Conversion, Tammannstr. 6, 37077 Göttingen, Germany

Mail: inke.siewert@chemie.uni-goettingen.de

Table of content

Instrumentation S3
Syntheses S4
Reduction of **2H** S4
H_2 quantification after electrochemical reduction of **1H** and $DBUH^+$ S5
General procedure for catalytic N_2 reduction S5
Procedure for catalytic N_2 reduction under $^{15}N_2$ S5
General procedure for the electrochemical N_2 splitting with $\mathbf{1}^-$ S5
Quantification by ^{31}P NMR spectroscopy S5
Quantification by ^{1}H NMR spectroscopy S6
Utilizing $^{15}N_2$ S6
UV/Vis Titration of **1H** S6
Electroanalytical data S8
CV and DPV data of **1H** S8
CV data of $\mathbf{I}^{\mathbf{Cl}}$ S11
CV data of **2H** S11
CV data of $\mathbf{3}^-$ S12
Electrochemical data of $DBUH^+$ S12
UV/Vis Spectroelectrochemistry of **1H** S13
IR Spectroelectrochemistry of **1H** S14
EPR spectra S15
NMR spectra S16
Data of the electrochemical N_2 splitting with $\mathbf{1}^-$ S19
Quantification of $\mathbf{3}^-$ by ^{31}P-NMR spectroscopy S19
Quantification of ammonium by ^{1}H NMR spectroscopy S22
IR spectra S22
Mass spectra S23
Quantification of H_2 ..... S26
X-Ray Crystallography S26

Instrumentation

^{1}H-, ^{13}C- and ^{31}P-NMR spectra were recorded with a Bruker Avance 300 or a Bruker Avance 400 NMR spectrometer with $CDCl_3$, CD_2Cl_2, THF-d_8 and DMSO-d_6 as the solvents at 25 °C. The ^{1}H-, ^{13}C-NMR spectra were calibrated against the residual protons and natural-abundance ^{13}C resonances of the deuterated solvents ($CDCl_3$: δ_H = 7.26 ppm, δ_C = 77.2 ppm; CD_2Cl_2: δ_H = 5.32 ppm, δ_C = 53.8 ppm; THF-d_8: δ_H = 1.72 and 3.58 ppm, δ_C = 67.2 and 25.3 ppm; DMSO-d_6: δ_H = 2.50 ppm, δ_C = 39.5 ppm). ^{31}P-NMR spectra are reported relative to external standard phosphoric acid (δ_P = 0.0 ppm). Signal multiplicities are reported as: s (singlet), d (doublet), t (triplet), q (quartet), m (multiplet), br (broad). The magnetic susceptibility of **1H** was determined by Evans' method and corrected for the diamagnetic contribution by using the approximation of half of the molecular weight of the substance.[1]

Microanalyses were performed with an Elementar Vario El II elemental analyser.

Mass spectra were recorded using a Bruker APEX IV micrOTOF or a Thermo Scientific LTQ Orbitrap XL mass spectrometer.

The IR spectra were recorded with a Bruker *Invenio-R* spectrometer. The IR-SEC experiments were conducted with an OTTLE cell[2] containing an Au-gauze working electrode, a Pt counter electrode, and an Ag pseudo reference electrode. Reductive sweep voltammograms were recorded with a scan rate of 0.0025 Vs^{-1} and IR spectra were recorded every 15 s.

The UV/Vis and UV/Vis-SEC data were recorded with a BWTek *Exepmplar LS* spectrometer with a BWTek Deuterium/Tungsten light source. For the UV/Vis-SEC experiments, a thin layer cuvette (0.1 cm) was used equipped with a Pt-gauze working electrode, a Pt counter electrode, and a silver wire in a sample holder with an electrolyte solution as pseudo reference electrode. A CV was recorded to determine the potentials, and the cell was re-filled. The desired potential was applied, and a UV/Vis spectrum was recorded every 10 s until the current dropped to the capacitive current.

EPR spectra of **2H** was recorded on a Bruker *ElexSys E500* X-band spectrometer equipped with the digital temperature control system ER 4131VT along with the ER 049 Microwave Bridge. The EPR spectra of **1H** was recorded on a Magnettech *Miniscope MS400* X-band spectrometer at ambient temperature. The *g*-value was determined through external calibration using a manganese(II) acetate reference sample at identical experimental parameters to that of the sample. For the specific experimental setting please see Figure S 16 and Figure S 17. Spectra simulation was performed using EasySpin v5.2.35 within Matlab (MathWorks) software suite.[3] Species recorded in fluid solution were assumed to be in the isotropic (fast-motional) regime. Their spectra were hence simulated utilizing the 'garlic' function.

H_2 was quantified with a Shimadzu GC-2014 equipped with a Thermal Conductivity Detector (TCD) and a 4 Å molecular sieve column. Methane was used as an internal standard in order to determine n_{H2}. A calibration curve for CH_4/H_2 was determined by preparing known quantities of the mixtures using the same set-up as in the electrolysis experiment and injecting to the chromatograph.

Syntheses

Reduction of **2H**

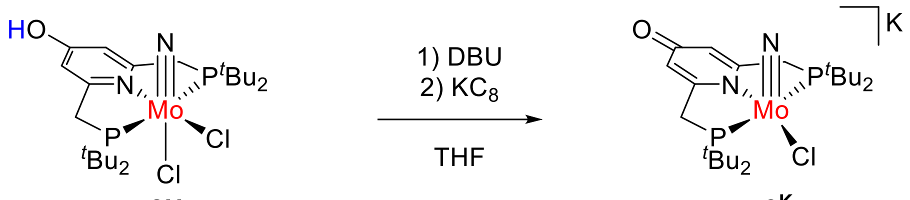

DBU (10.3 mg, 67.5 µmol, 1.00 equiv.) was added to a solution of **2H** (40.0 mg, 67.5 µmol, 1.00 equiv.) in THF (3 mL) and stirred for 5 min. KC_8 (30.1 mg, 223 µmol, 3.30 equiv.) was added and the mixture was further stirred for 2 h at room temperature. The mixture was filtered, and the solution was dried *in vacuo*. The residue was washed with hexane (3 x 1 mL) and Et_2O (5 x 1 mL) and dried *in vacuo* to afford **3**$^{\mathbf{K}}$ as a brown solid. The sample contained ca. 1 equiv. of L^- as a side product.

Reaction of **2H** with 4.40 equiv. of KC_8 also afforded **3**$^{\mathbf{K}}$ as monitored by ^{31}P-NMR spectroscopy with significantly higher amounts of L^- as a side product (ratio **3**$^{\mathbf{K}}$/L^- ca. 1/5).

^{1}H{^{31}P}-NMR (400 MHz, THF-d_8) δ (ppm) = 6.13 (s, <u>H</u>$^{3\text{-Py}}$, 2H), 3.13 (s, C<u>H</u>$_2$, 4H), 1.28 (s, PC(C<u>H</u>$_3$)$_3$, 36H). ^{13}C{^{1}H}-NMR (100 MHz, THF-d_8) δ (ppm) = 113.0 (<u>C</u>$^{3\text{-Py}}$), 38.5 (<u>C</u>(CH$_3$)$_3$), 36.3 (<u>C</u>H$_2$), 30.1 (C(<u>C</u>H$_3$)$_3$), signals of the quaternary carbon atoms of the pyridone moiety were not observed. ^{31}P{^{1}H}-NMR (162 MHz, THF-d_8) δ (ppm) = 85.1 (s).

IR (ATR): $\tilde{v}$ (cm^{-1}) = 2962 (m), 2900 (m), 2864 (m), 1581 (s), 1508 (w), 1471 (m), 1365 (w), 1261 (m), 1161 (w), 1094 (m), 1020 (m), 843 (w), 802 (m).

The reduction of **1H** (9.3 mg, 15.2 µmol, 1.00 equiv.) with DBU (2.3 mg, 15.2 µmol, 1.00 equiv.) and ex. of KC_8 (11.3 mg, 83.3 µmol, 5.5 equiv.) in THF (2 mL) led to the formation of the same species as reasoned from ^{31}P-NMR spectroscopy.

NH_3 quantification from **2H/2**$^-$

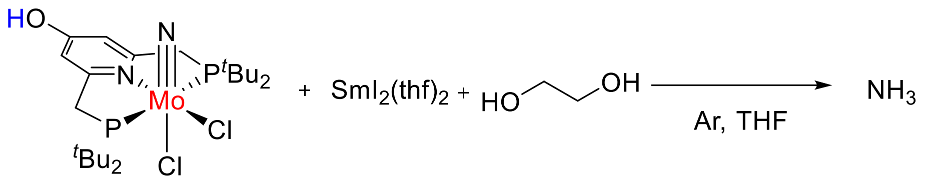

2H

All manipulations were conducted under Ar atmosphere. A solution of ethylene glycol (2.08 µL, 37.1 µmol, 10.0 equiv.) in THF (5 mL) was prepared and degassed by five freeze-pump-thaw cycles. The solution was transferred to a Schlenk flask containing **2H** (2.20 mg, 3.71 µmol, 1.00 equiv.) and $SmI_2(thf)_2$ (21.9 mg, 37.1 µmol, 10.0 equiv.). The solution was stirred overnight, frozen and an excess of KO^{t}Bu (~ 30 mg) in MeOH (0.5 mL) was added to the frozen solution. The solution was allowed to warm to rt and stirred for 15 min. All volatiles were vacuum transferred into a liquid-N_2-cooled Schlenk flask containing HCl (2 M in Et_2O, 3 mL). The solution was stirred at rt for 15 min, volatiles were removed *in vacuo*, and the residue was dissolved in DMSO-d_6 with a trimethoxybenzene as an internal standard. The amount of ammonium (3.1 µmol, 85%) was determined by integration vs the internal standard.

The same procedure was utilized to determine the amount of ammonium by ^{1}H NMR spectroscopy after electrolysis of **1**$^-$.

H$_2$ quantification after electrochemical reduction of **1H** and DBUH$^+$

Controlled potential electrolysis was performed in a custom-made H-type cell with a P3 glass frit to separate working and counter chamber. The working chamber was equipped with a 3 mm diameter glassy carbon rod (ALS Japan) and a silver wire in electrolyte solution in a fritted sample holder as pseudo reference electrode. The counter chamber was equipped with a Pt spiral counter electrode. Both chambers were filled with 3 mL electrolyte solution (0.2 M ^{n}Bu$_4$NPF$_6$ in THF) and a stirring bar was added. **1H** (4.9 mg, 8.0 µmol) or [DBUH]Cl (2.1 mg, 11.1 µmol) was added to the working chamber. A potential of −2.1 V for **1H** and −2.6 V for [DBUH]Cl was applied. The solution of **1H** was electrolyzed until 1 charge equivalent was passed. The solution of [DBUH]Cl was electrolyzed until the current dropped down to approximately 10% of the initial current. Subsequently, methane was injected in the cell as internal standard and the gas phases were analyzed by GC-TCD.

General procedure for catalytic N$_2$ reduction

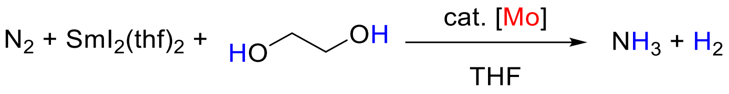

Dry THF (6 mL) was added to the catalyst (2.00 µmol, 1.00 equiv.) and SmI$_2$(thf)$_2$ (197 mg, 360 µmol, 180 equiv.) in a N$_2$ filled glovebox. Ethylene glycol (22.4 mg, 360 µmol, 180 equiv.) was added in one portion and the mixture was further stirred for 18 h at rt resulting in a colour change from deep blue to yellow. The mixture was frozen to −196 °C and an excess of KO^{t}Bu (~ 100 mg) in MeOH (5 mL) was added. The mixture was allowed to warm to rt and stirred for 15 min. The volatiles were vacuum transferred into a liquid N$_2$ cooled Schlenk flask containing HCl (2 M in Et$_2$O, 3 mL), The solution was stirred for further 15 min at rt, the solvents were removed *in vacuo* and the residue was dissolved in DMSO-d_6 with trimethoxybenzene as an internal standard. The amount of ammonium was determined by integration vs the internal standard.

Procedure for catalytic N$_2$ reduction under ^{15}N$_2$

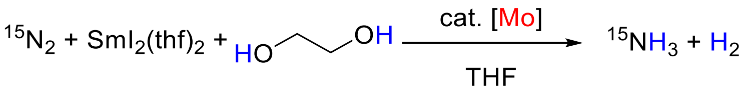

A solution of ethylene glycol (22.4 mg, 360 µmol, 180 equiv.) in THF (6 mL) was degassed by five freeze-pump-thaw cycles and transferred to a 2-necked round bottom flask containing the catalyst (2.00 µmol, 1.00 equiv.) and SmI$_2$(thf)$_2$ (197 mg, 360 µmol, 180 equiv.). The solution was frozen with liquid N$_2$. The headspace was evacuated and replaced by ^{15}N$_2$. The flask was sealed, and the solution was warmed to room temperature. The solution was stirred overnight, and the amount of ammonium was quantified as described above.

General procedure for the electrochemical N$_2$ splitting with **1**$^-$

Quantification by ^{31}P NMR spectroscopy

Controlled potential electrolyses were performed in a custom-made H-type cell with a P3 glass frit to separate working and counter chamber under N$_2$ atmosphere. The working chamber was equipped with a 7 mm diameter glassy carbon rod (ALS Japan) and a silver wire in electrolyte solution in a fritted sample holder as pseudo reference electrode. The counter chamber was equipped with a Zn rod as sacrificial electrode. Both chambers were filled with 2.5 mL electrolyte solution (0.2 M ^{n}Bu$_4$NPF$_6$ in THF) and a stirring bar was added. **1H** (12.3 mg, 20 µmol) and DBU (3.00 µL, 3.04 mg, 20 µmol) were added to the working chamber. A fixed potential was applied, and the solution was electrolyzed until a desired amount of charge was passed. An aliquot of the solution in the working chamber was taken and a drop of THF-d_8 was added. The mixture was analyzed by ^{31}P-NMR spectroscopy, and **3**$^-$ was quantified by integration vs the PF$_6^-$ anion.

Quantification by ^{1}H NMR spectroscopy

The electrolysis was conducted as described above. After electrolysis, the solutions of both chambers were transferred into a Schlenk flask and the cell washed two times with THF. The solution was dried *in vacuo* and $SmI_2(thf)_2$ (110 mg, 200 µmol, 10.0 equiv.) was added under Ar atmosphere. Ethylene glycol (11.2 µL, 12.4 mg, 200 µmol, 10.0 equiv.) in THF (10 mL) was degassed in five consecutive freeze-pump-thaw cycles and added to the residue. The same procedure as for the detection of ammonia in **2H** was utilized to quantify ammonium by ^{1}H NMR spectroscopy.

Utilizing $^{15}N_2$

A H-type cell with NS14 joint connection containing **1H** (12.3 mg, 20 µmol) and DBU (3.00 µL, 3.04 mg, 20 µmol) in the working chamber was equipped with the electrodes. The electrolyte solution (0.2 M $^{n}Bu_4NPF_6$ in THF, 5 mL) was degassed in five consecutive freeze-pump-thaw cycles and added to the cell (2.5 mL per chamber). The solution was frozen in liquid N_2, the cell was evacuated, and $^{15}N_2$ was added. The solution was warmed to rt and electrolysis was started at a fixed potential. After injection of 3 charge equivalents, the electrolysis the cell was transferred into a glovebox and work up was conducted as described in the experiment using $^{14}N_2$.

UV/Vis Titration of **1H**

2 mL of a stock solution of **1H** in THF (0.15 mM) were added into a quartz cuvette (1 cm diameter) and a UV/Vis spectrum recorded. A solution of DBU in THF (15 mM, 4 µL, 0.2 equiv.) was added sequentially in 0.2 equiv. steps recording UV/Vis spectrum after each addition until 2 equiv. of DBU were added. Back titration with a solution of LutHOTf in THF (15 mM, 4 µL, 0.2 equiv.) was done analogously in 0.2 equiv. until 2 equiv. of LutHOTf were added. The spectra are shown in Figure S 1.

Fitting of the data as described in Ref. 4 led to the pK_a of **1H**.

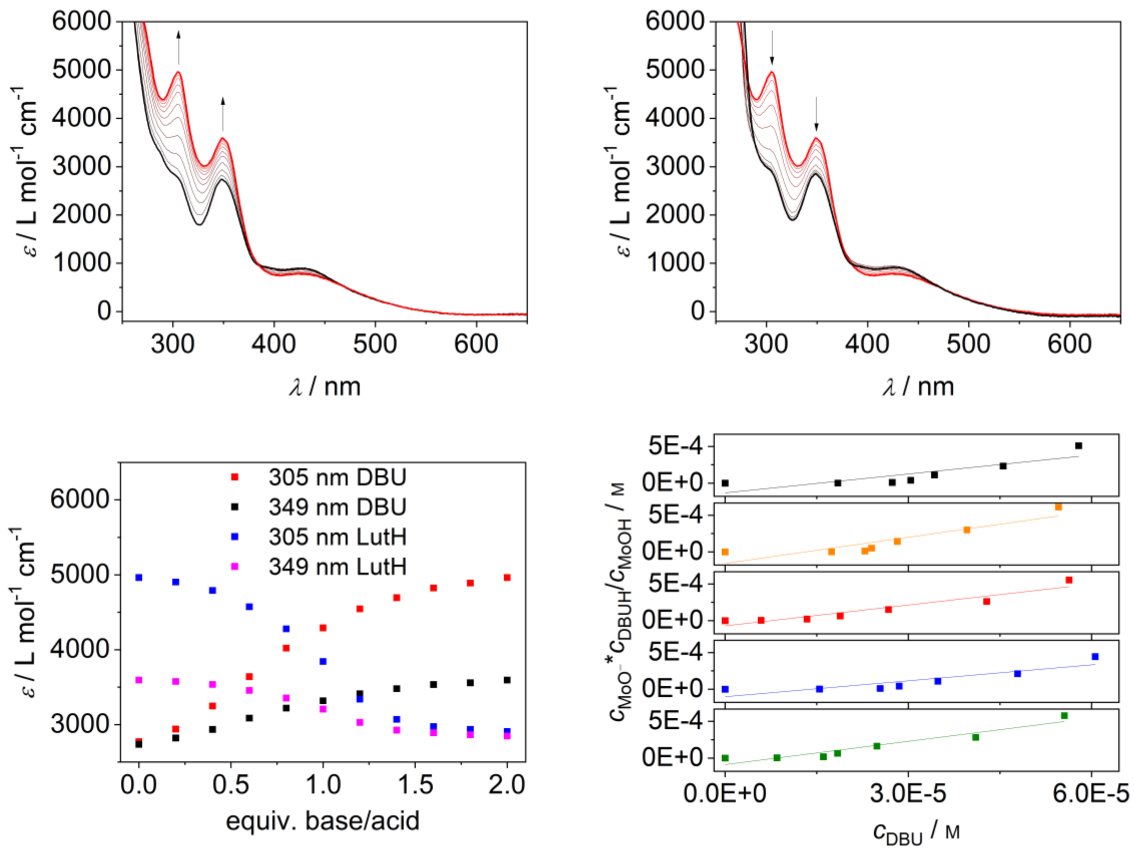

Figure S 1. *Top left: UV/Vis spectra during the titration of* ***1H*** *(0.15 mM) with 2 equiv. DBU, black: beginning, red: end. Top right: backtitration of* ***1H*** *and 2 equiv. DBU with 2 equiv. lutidinium, red: beginning, black: end. Bottom left: extinction at 305 nm and 349 nm during the gradual addition of DBU and lutidinium. Bottom right:* K_{eq} *determination from the titration with DBU at different wavelengths: 305 nm (black), 310 nm (orange), 340 nm (red), 350 nm (blue), 360 nm (green). All data in THF.*

Table S 1. K_{eq} *determined at different wavelengths from the titration experiment of* ***1H*** *with DBU.*

**wavelength / nm**	K_{eq}
305	8.57
310	11.8
340	9.45
350	7.23
360	10.5

Electroanalytical data

CV and DPV data of **1H**

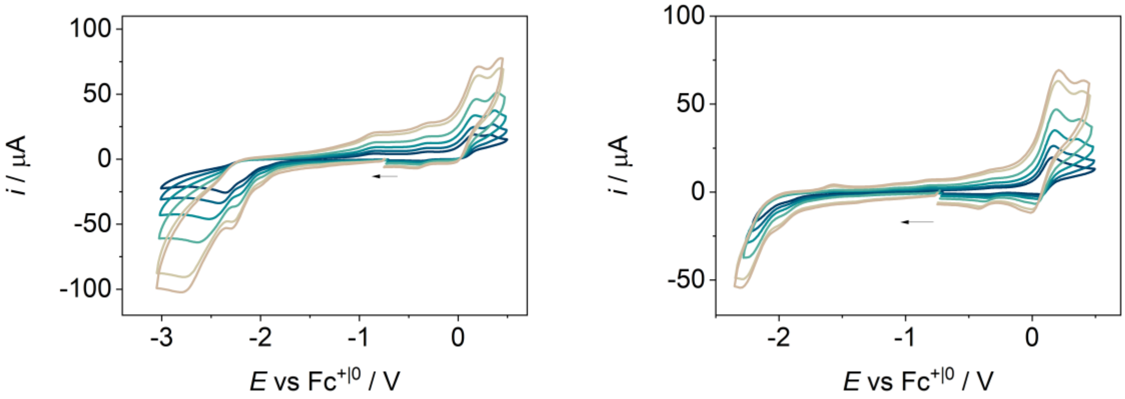

Figure S 2. *Scan rate dependent CV data of* ***1H*** *with different return potentials, THF,* $c_{\mathbf{1H}}$ *~ 1 mM, I = 0.2 M* $^{n}Bu_4NPF_6$*, v = 0.05, 0.1, 0.2, 0.4, 0.8, and 1* Vs^{-1}*, Ar atmosphere.*

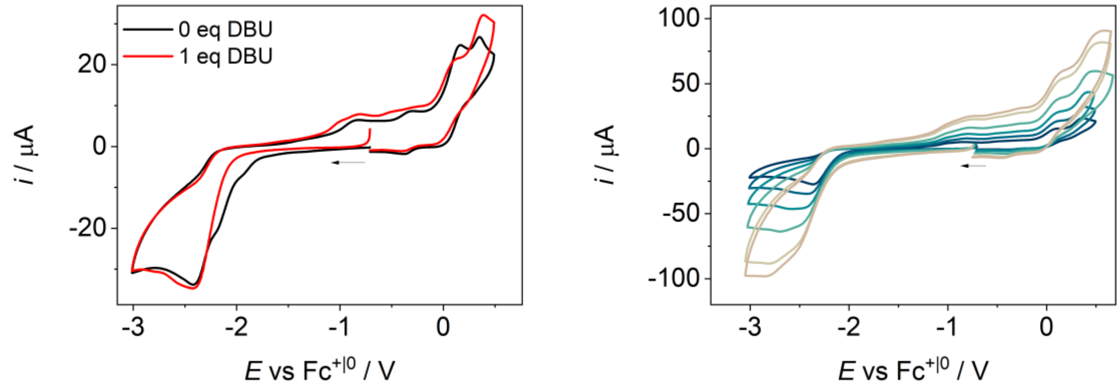

Figure S 3. *Left: CV data of* ***1H*** *with 0 and 1 equiv. DBU, v = 0.1* Vs^{-1}*. Right: Scan rate dependent CV data of* ***1H*** *with 1.0 equiv. DBU, v = 0.05, 0.1, 0.2, 0.4, 0.8, and 1* Vs^{-1}*. Both THF, c ~ 1 mM, I = 0.2 M* $^{n}Bu_4NPF_6$*, Ar atmosphere.*

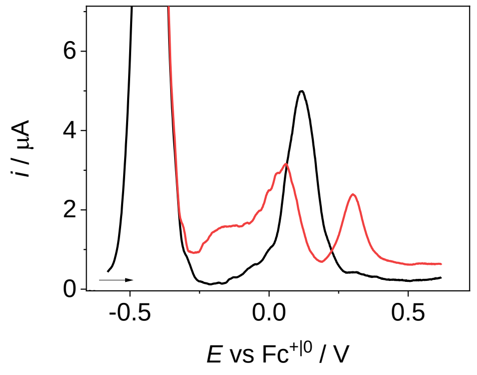

Figure S 4. *DPV data of* ***1H*** *with 0 (black) and 1 equiv. (red) of DBU, v = 0.002* Vs^{-1}*, pulse size 0.025 V, pulse time 0.1 s, sample period 1 s, THF, c ~ 1.5 mM, I = 0.2 M* $^{n}Bu_4NPF_6$*,* N_2 *atmosphere. The redox process at −0.43 V belongs to the* $Fc^{*+|0}$ *redox couple (standard).*

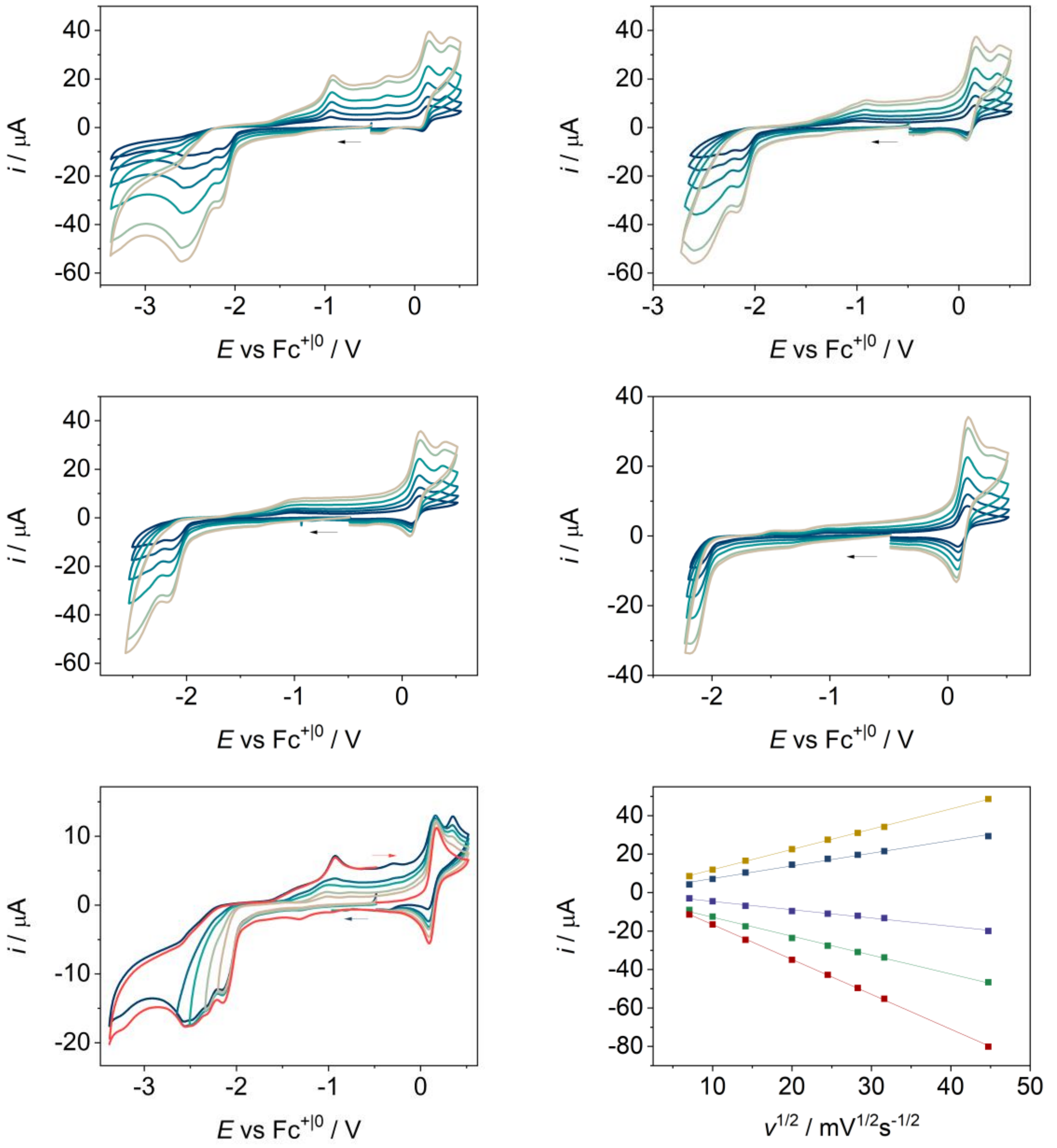

Figure S 5. *Scan rate dependent CV data of* ***1H*** *with different return potentials, v = 0.05, 0.1, 0.2, 0.4, 0.8, and 1 Vs^{-1}, bottom left: CV data of* ***1H*** *at 0.1 Vs^{-1}. All CV data are recorded in THF, $c_{\mathbf{1H}}$ ~ 0.5 mM, I = 0.2 M $^{n}Bu_4NPF_6$, N_2 atmosphere. Bottom right: square root of the scan rate vs current for the redox events at 0.17 V (yellow), −0.93 V (blue), 0.08 V (purple), −2.15 V (green) and −2.57 V (red).*

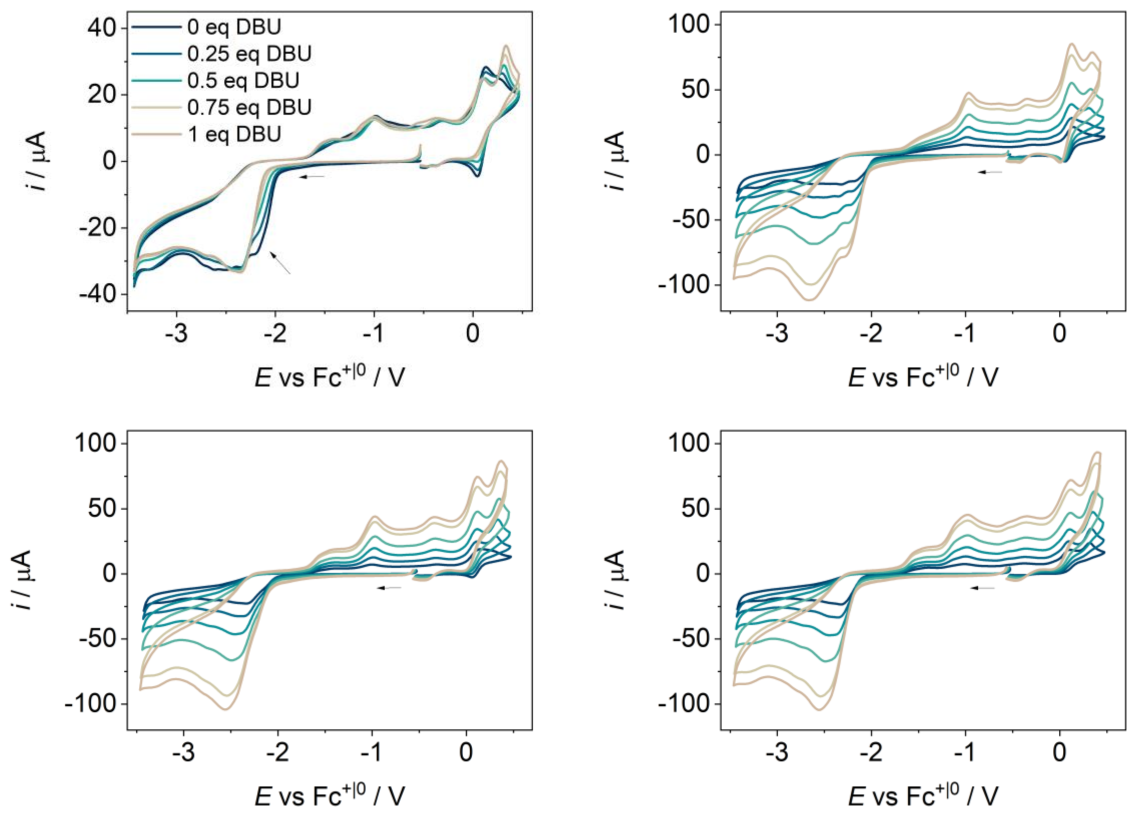

Figure S 6. *Top left: CV data of* ***1H*** *with various amounts of DBU, $v = 0.1$ Vs^{-1}. Others: scan rate dependent CV data of* ***1H*** *with 0.0 equiv. DBU (top right), 0.5 equiv. DBU (bottom left) and 1.0 equiv. (bottom right), $v = 0.05, 0.1, 0.2, 0.4, 0.8$, and 1 Vs^{-1}. All CV data are recorded in THF, $c_{\mathbf{1H}} \sim 1$ mM, $I = 0.2$ M ^{n}Bu$_4$NPF$_6$, N_2 atmosphere.*

CV data of **I**^{Cl}

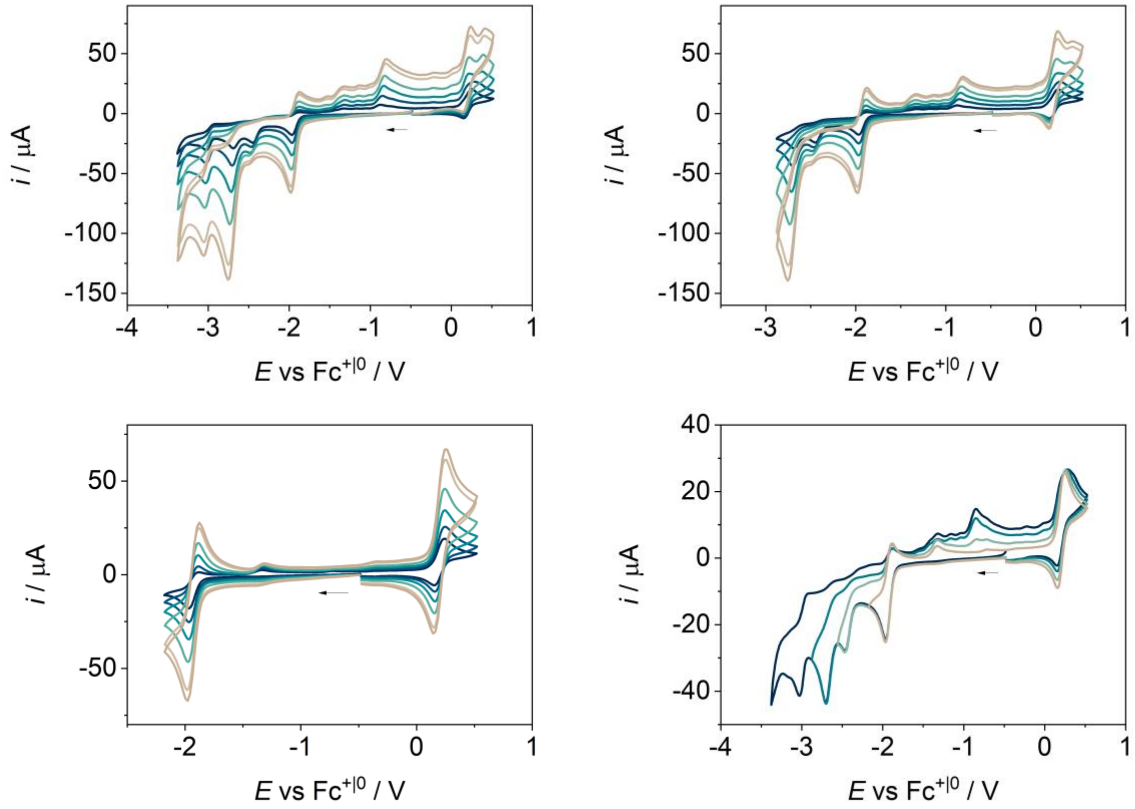

Figure S 7. *Scan rate dependent CV data of* ***I***^{Cl} *with different return potentials,* ν *= 0.05, 0.1, 0.2, 0.4, 0.8, and 1 Vs*$^{-1}$*, bottom right: CV data of* ***I***^{Cl} *at 0.1 Vs*$^{-1}$*. All CV data are recorded in THF,* $c_{\mathbf{ICl}}$ *~ 0.5 mM, I = 0.2 M* $^{n}Bu_4NPF_6$*,* N_2 *atmosphere.*

CV data of **2H**

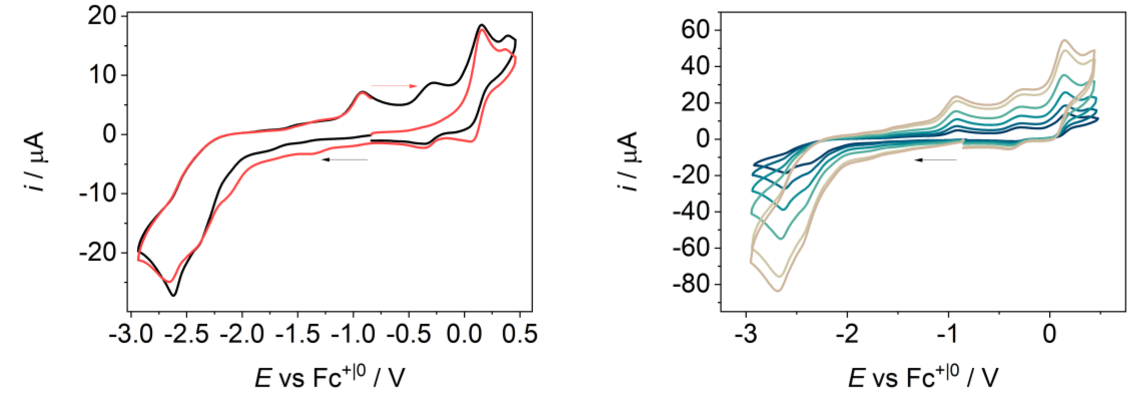

Figure S 8. *Left: CV data of* ***2H****,* ν *= 0.1 Vs*$^{-1}$*. Right: Scan rate dependent CV data of* ***2H****,* ν *= 0.05, 0.1, 0.2, 0.4, 0.8, and 1 Vs*$^{-1}$*. Both THF,* $c_{\mathbf{2H}}$ *~ 1 mM, I = 0.2 M* $^{n}Bu_4NPF_6$*,* N_2 *atmosphere.*

CV data of **3**$^{-}$

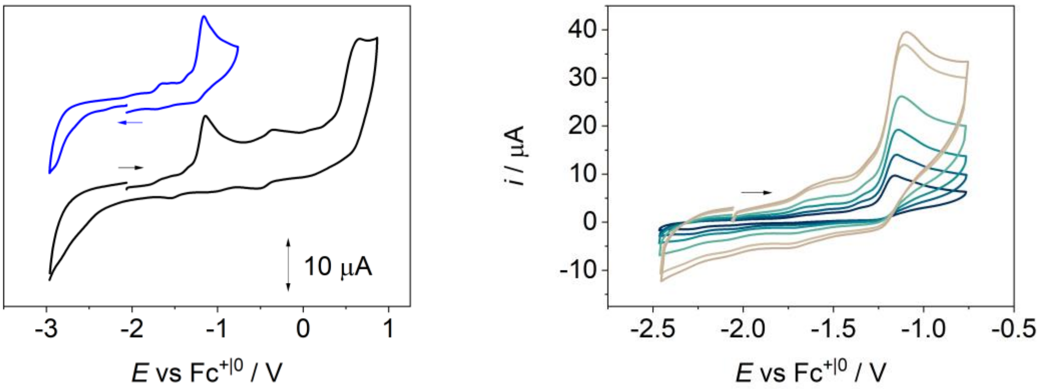

Figure S 9. *Left: CV data of* ***3***$^{-}$*, v = 0.1 Vs^{-1}. Right: Scan rate dependent CV data of* ***3***$^{-}$*, v = 0.05, 0.1, 0.2, 0.4, 0.8, and 1 Vs^{-1}. Both THF, $c_{\mathbf{3K}}$ ~ 1.5 mM, I = 0.2 M $^{n}Bu_4NPF_6$, N_2 atmosphere.*

Electrochemical data of DBUH^{+}

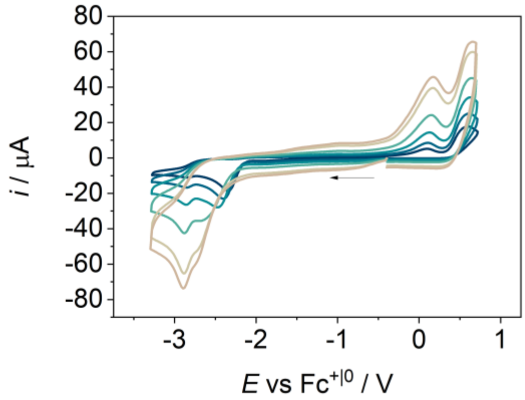

Figure S 10. *Scan rate dependent CV data of [DBUH]Cl, v = 0.05, 0.1, 0.2, 0.4, 0.8, and 1 Vs^{-1}, THF, c_{DBUH+} ~ 2 mM, I = 0.2 M $^{n}Bu_4NPF_6$, N_2 atmosphere.*

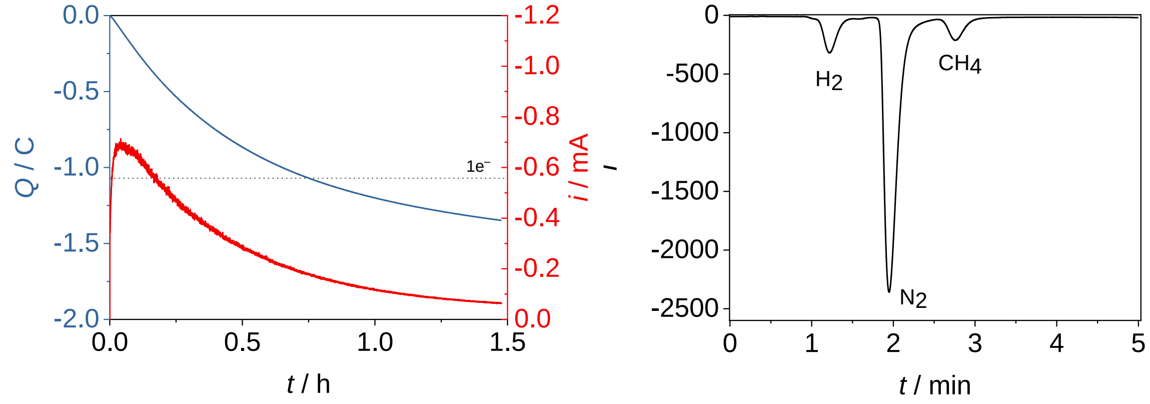

Figure S 11. *Left: Current (red) and charge (blue) over time plot of the CPE experiment of [DBUH]Cl in THF, $n_{[DBUH]Cl}$ = 14.8 µmol, I = 0.2* M *$^{n}Bu_4NPF_6$, E ~ −2.6 V vs $Fc^{+|0}$, N_2 atmosphere. Right: GC-TCD trace of the headspace of the electrolysis cell after the CPE experiment.*

UV/Vis Spectroelectrochemistry of **1H**

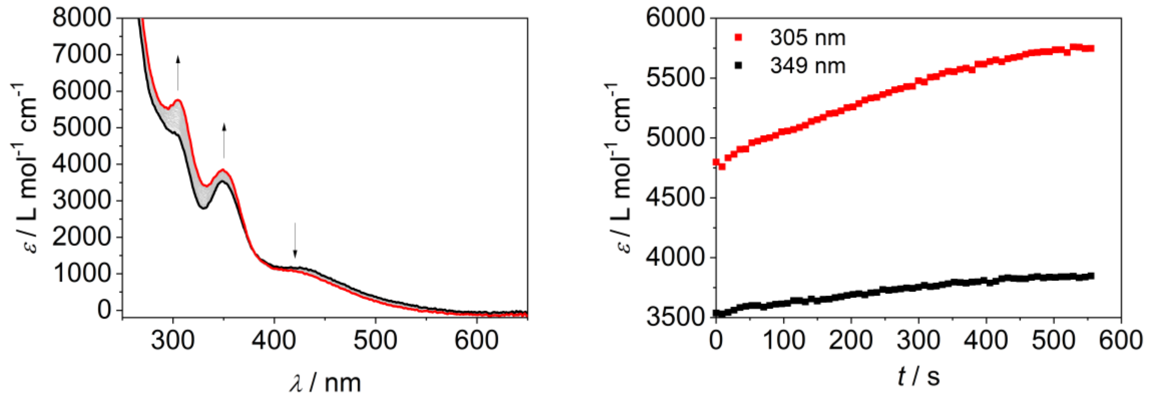

Figure S 12*. Left: UV/Vis-SEC of* ***1H****, THF, c_{1H} ~ 0.5 m*M*, I = 0.2* M *$^{n}Bu_4NPF_6$, N_2 atmosphere, E ~ −2.4 V vs $Fc^{+|0}$, black: beginning, red: end; Right: extinction at 305 and 349 nm during reduction.*

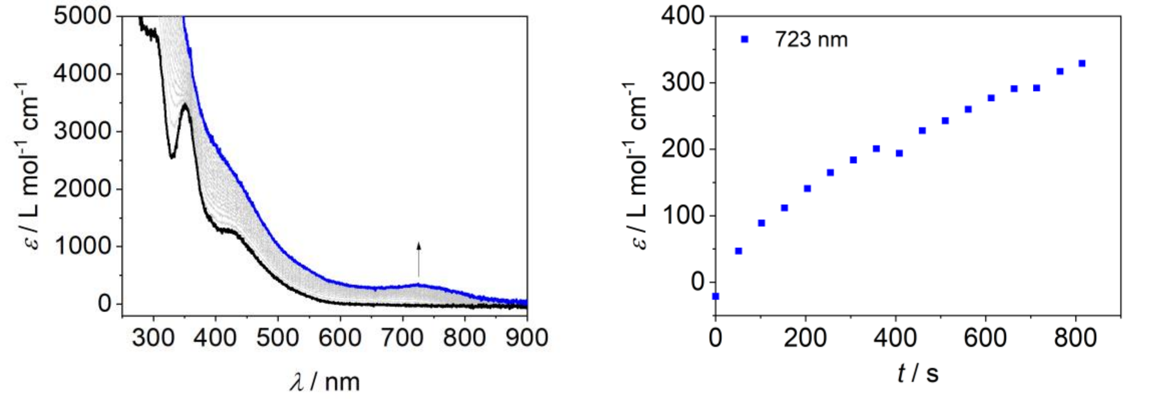

Figure S 13. *Left: UV/Vis-SEC of* ***1H****, THF, c_{1H} ~ 0.5 m*M*, I = 0.2* M *$^{n}Bu_4NPF_6$, N_2 atmosphere, E ~ −2.7 V vs $Fc^{+|0}$, N_2 atmosphere, black: beginning, blue: end; Right: extinction at 723 nm during reduction.*

IR Spectroelectrochemistry of **1H**

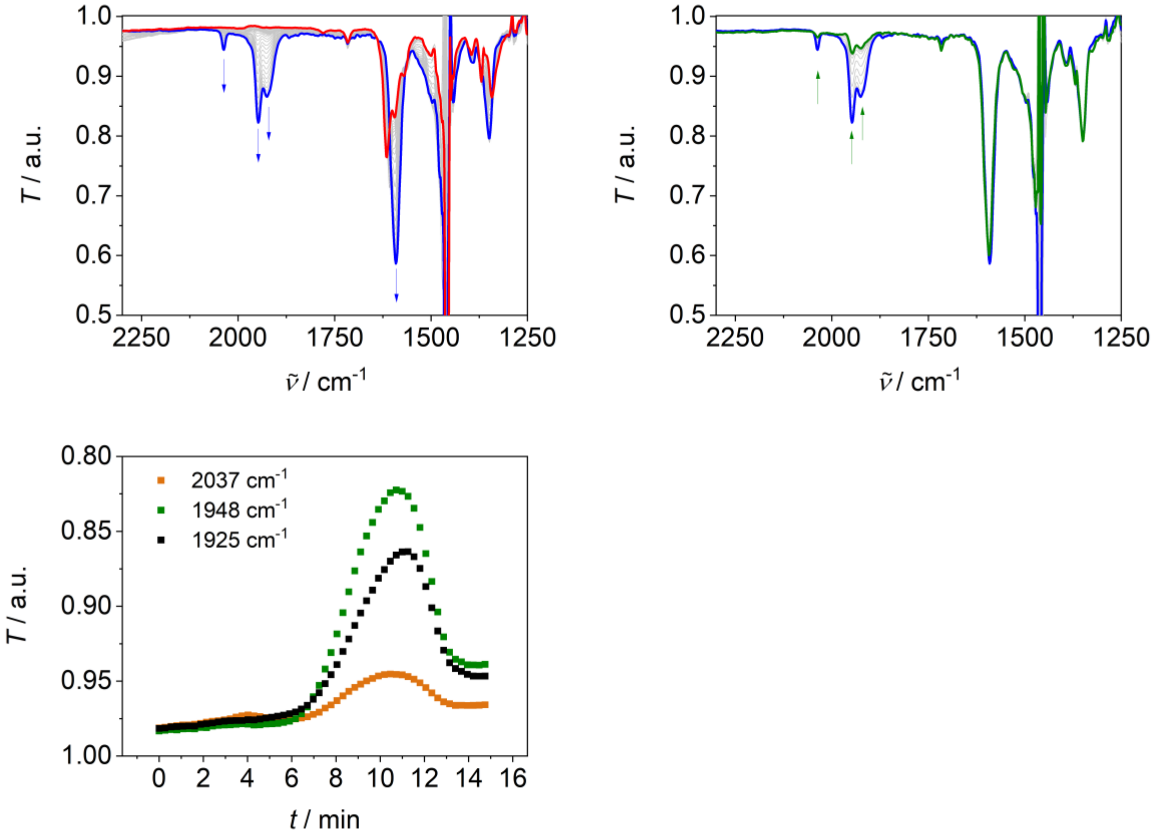

Figure S 14. *Top: IR-SEC spectra upon running a reductive CV of* ***1H****, ν = 0.0025 Vs^{-1}, THF, I = 0.2* м *^{n}Bu$_4$NPF$_6$, N$_2$ atmosphere; initial spectrum red, followed by blue, and green spectra; bottom: transmission of the terminal N≡N vibrations during the experiment.*

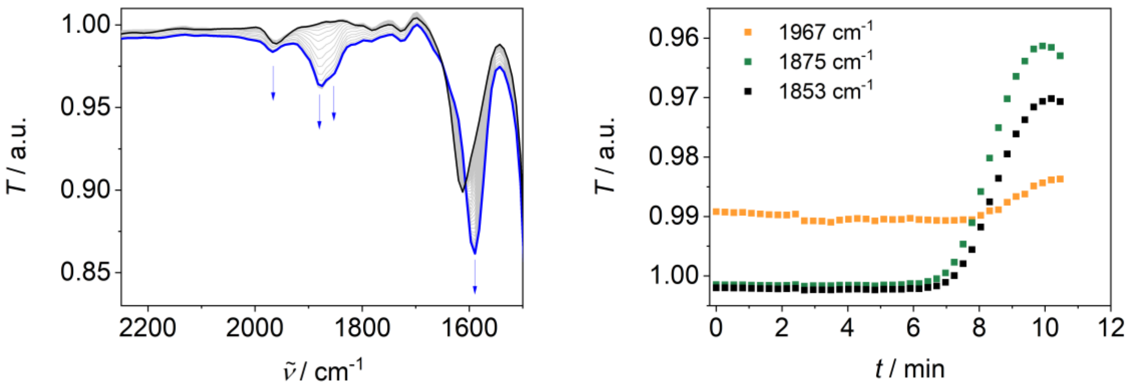

Figure S 15. *Left: IR-SEC spectra upon running a reductive CV of* ***1H****, ν = 0.0025 Vs^{-1}, THF, I = 0.2* м *^{n}Bu$_4$NPF$_6$, ^{15}N$_2$ atmosphere; initial spectrum black, end blue; right: transmission of the terminal ^{15}N≡^{15}N vibrations during the experiment.*

EPR spectra

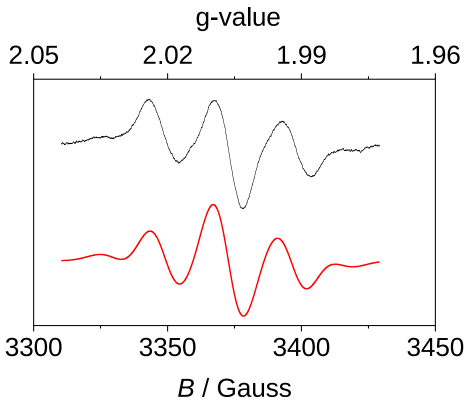

Figure S 16. *X-band EPR spectrum (black) of* ***1H*** *recorded in a THF solution at ~ 293 K. Microwave frequency of about 9.5 GHz, power 10 mW, field modulation amplitude 6 G, power attenuation 10 dB. Simulated spectrum (red); simulation parameters: S = 3/2;* g_{iso} *= 2.0093; A(2×*^{31}*P) = 67 MHz, A(1×*$^{95/97}$*Mo) = 76 MHz; isotropic line broadening (Gaussian, FWHM) lw = 0.21.*

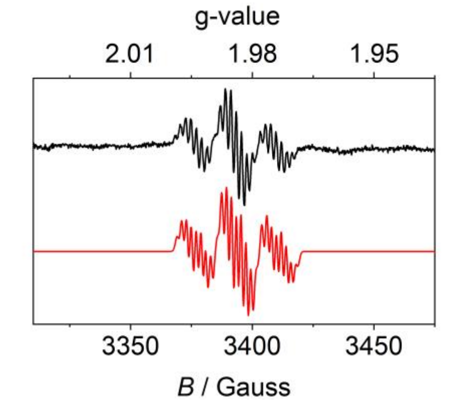

Figure S 17. *X-band EPR spectrum (black) of* ***2H*** *recorded in a THF solution at ~ 279 K. Microwave frequency of about 9.4 GHz, power 10 mW, field modulation amplitude 1 G, power attenuation 13 dB. Simulated spectrum (red); simulation parameters: S = 1/2;* g_{iso} *= 1.9849; A(2×*^{31}*P) = 40.6 MHz; A(2×*^{1}*H) = 4.2 MHz; A(1×*^{14}*N) = 9.8 MHz; A(1×*^{14}*N) = 4.5 MHz; isotropic line broadening (Gaussian, FWHM) lw = 0.17.*

NMR spectra

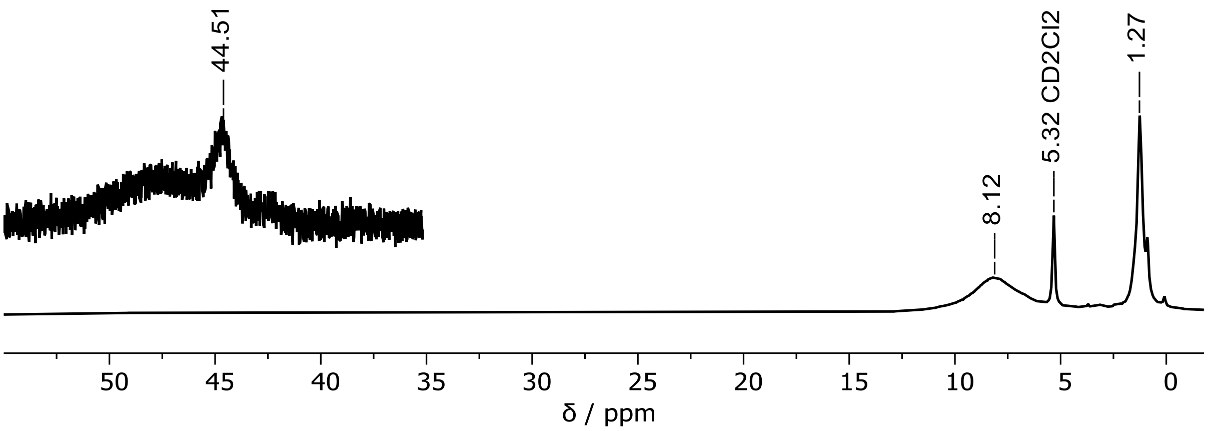

Figure S 18. *^{1}H-NMR spectrum of **1H** in CD_2Cl_2.*

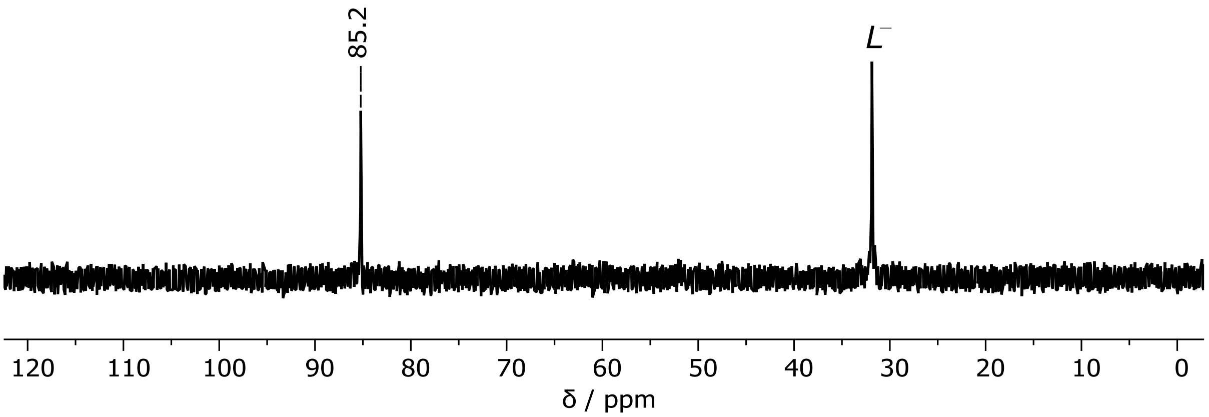

Figure S 19. *$^{31}P\{^1H\}$-NMR spectrum of $\mathbf{3^K}$ in THF-d_8.*

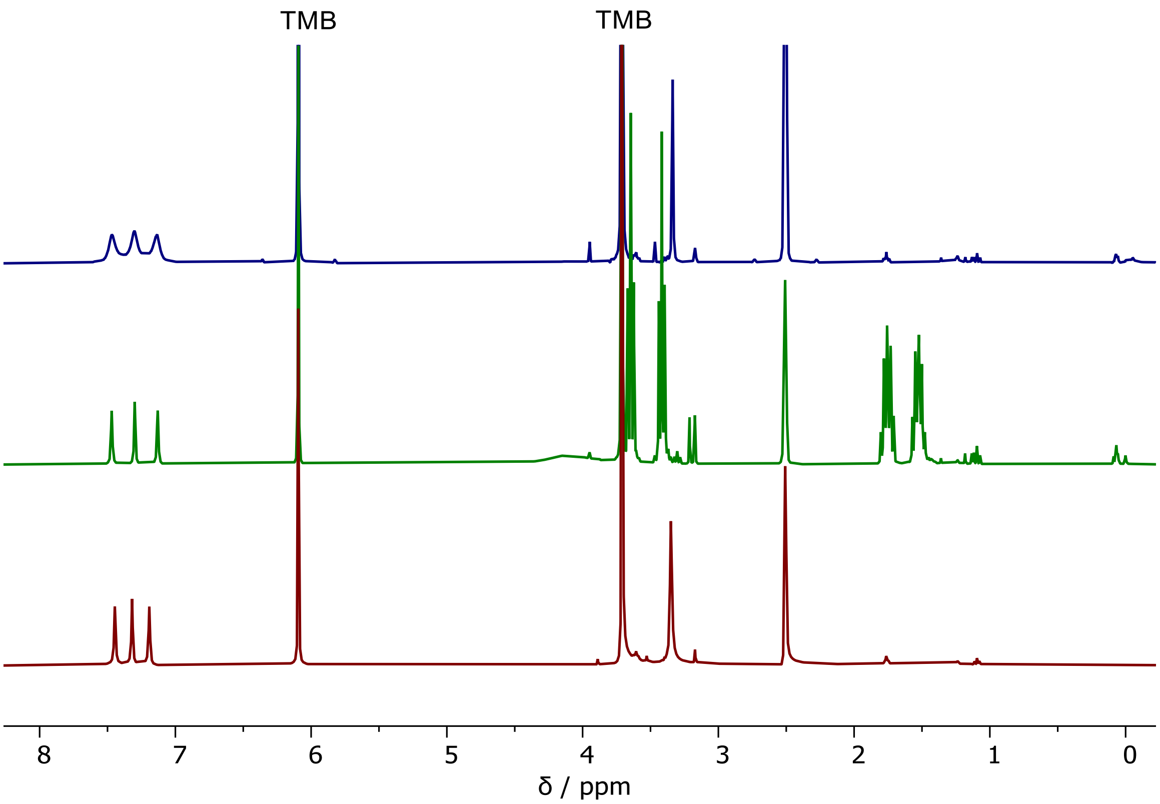

Figure S 20. *^{1}H-NMR-spectrum of NH_4^+ generated by N_2 reduction catalyzed by* ***1H****, DMSO-d$_6$, internal standard 1,3,5-trimethoxybenzene (TMB), three independent runs.*

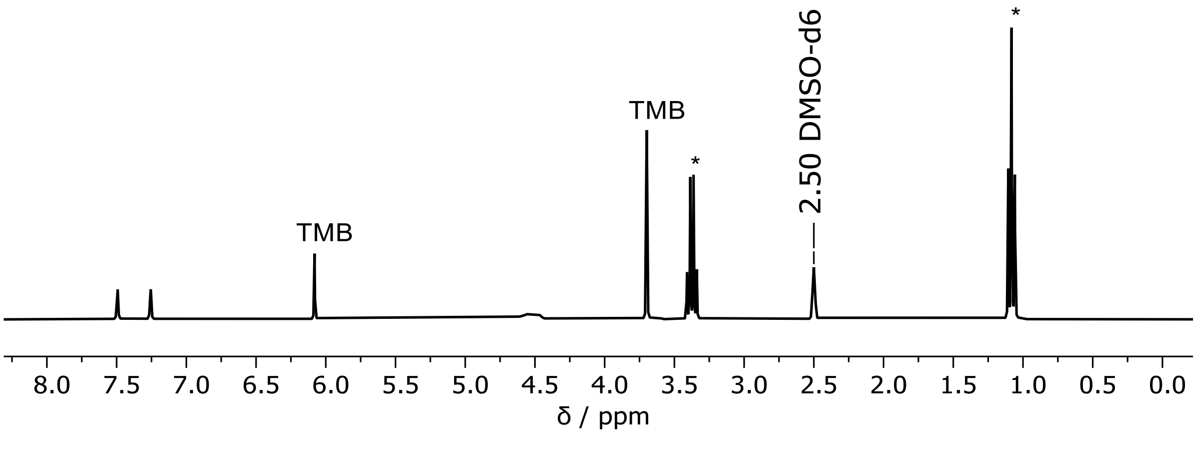

Figure S 21. *^{1}H-NMR-spectrum of $^{15}NH_4^+$ generated by $^{15}N_2$ reduction catalyzed by* ***1H****, DMSO-d$_6$, internal standard 1,3,5-trimethoxybenzene (TMB), diethyl ether marked with asterisks.*

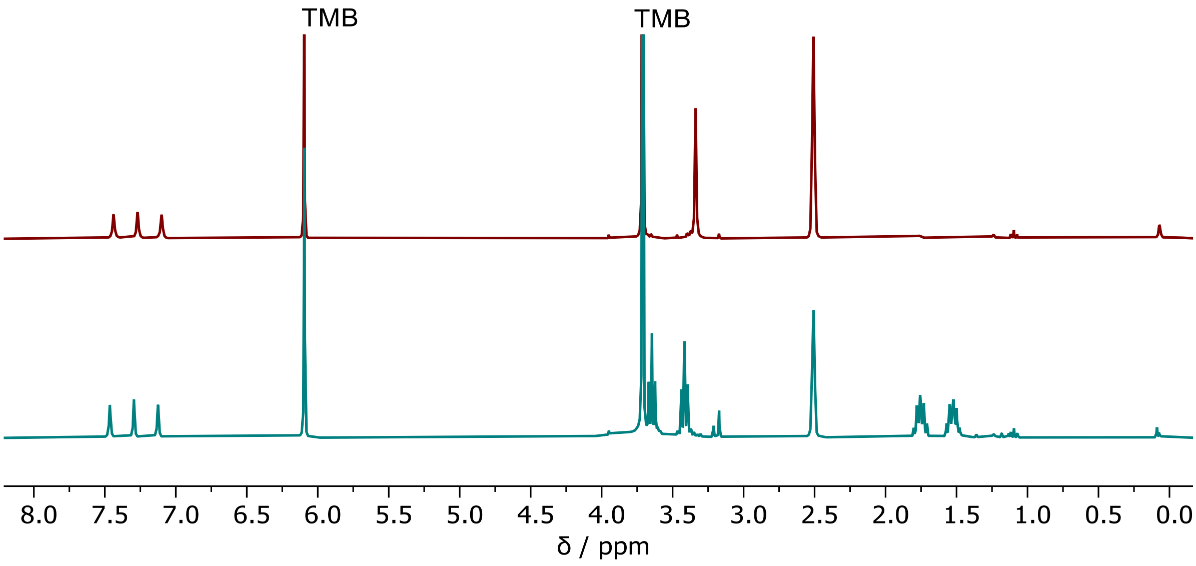

Figure S 22. *^{1}H-NMR-spectrum of NH_4^+ generated by N_2 reduction catalyzed by **2H**, DMSO-d$_6$, internal standard 1,3,5-trimethoxybenzene (TMB), two independent runs.*

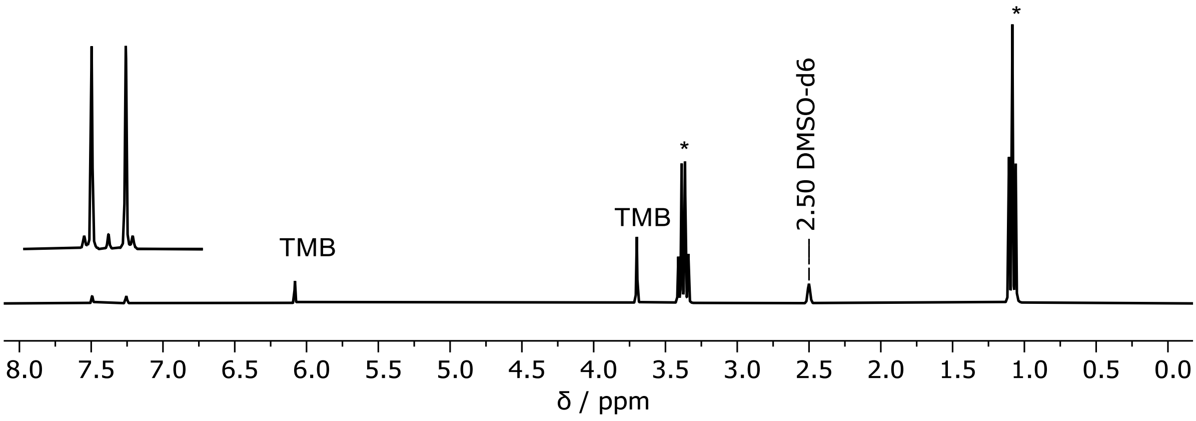

Figure S 23. *^{1}H-NMR-spectrum of $^{15}NH_4^+$ and 1 equiv. $^{14}NH_4^+$ generated by $^{15}N_2$ reduction catalyzed by **2H**, internal standard 1,3,5-trimethoxybenzene (TMB), diethyl ether marked with asterisks.*

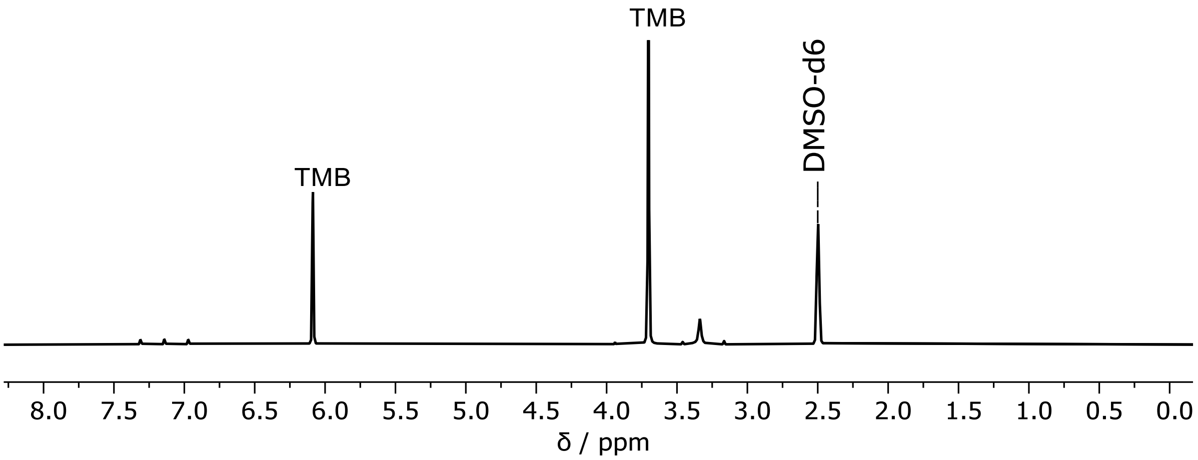

Figure S 24. *^{1}H-NMR-spectrum of NH_4^+ released from* ***2H*** *by adding 10 equiv. $SmI_2(thf)_2$ and 10 equiv. ethylene glycol under Ar, internal standard 1,3,5-trimethoxybenzene (TMB).*

Data of the electrochemical N_2 splitting with **1**$^-$

Quantification of **3**$^-$ by ^{31}P-NMR spectroscopy

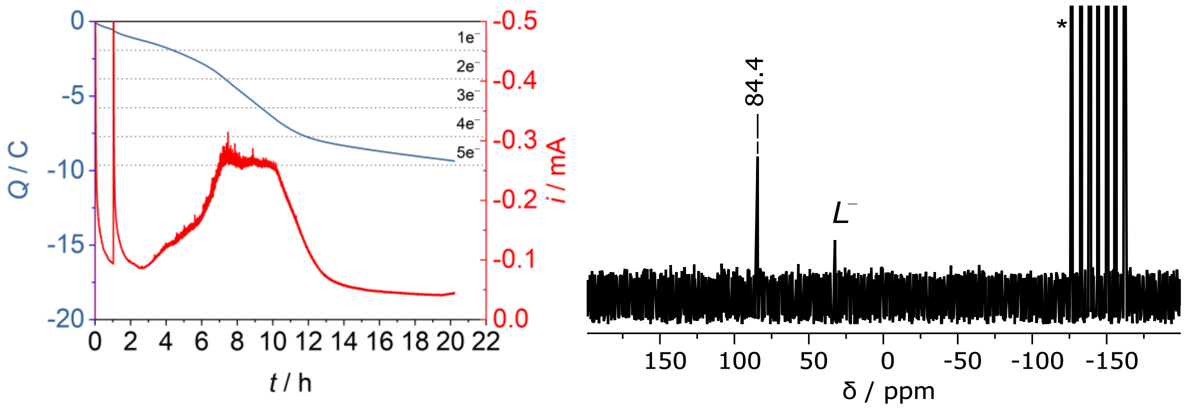

Figure S 25. *Left: Current (red) and charge (blue) over time plot of the CPE experiment of* ***1H*** *with 1 equiv. DBU in THF, $n_{\mathbf{1H}}$ = 20 µmol, I = 0.2 M ^{n}Bu$_4$NPF$_6$, E = −2.69 V vs Fc$^{+|0}$, N_2 atmosphere. Right: ^{31}P-NMR spectrum of the resulting solution in the working chamber; ^{n}Bu$_4$NPF$_6$ marked with an asterisk.*

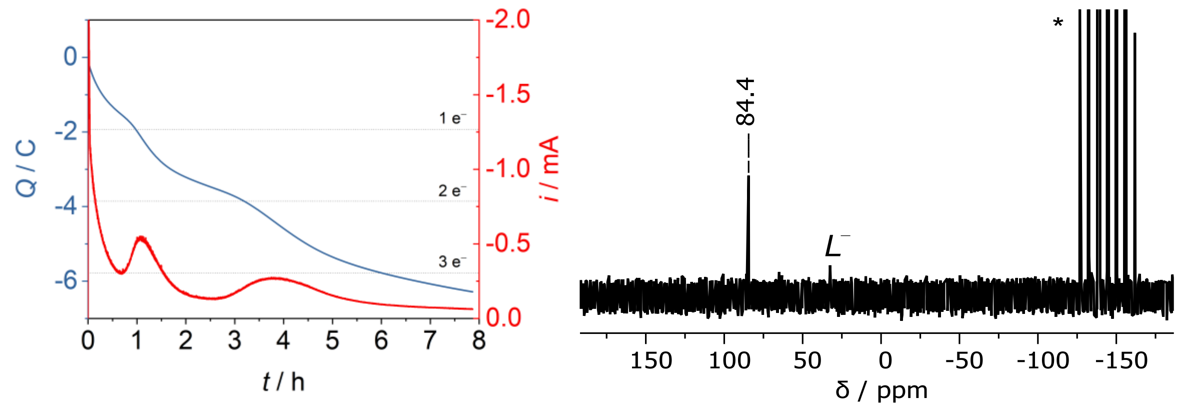

Figure S 26. *Left: Current (red) and charge (blue) over time plot of the CPE experiment of* ***1H*** *with 1 equiv. DBU in THF, $n_{\mathbf{1H}}$ = 20 µmol, I = 0.2* M *$^{n}Bu_4NPF_6$, E = −2.93 V vs $Fc^{+|0}$, N_2 atmosphere. Right: ^{31}P-NMR spectrum of the resulting solution in the working chamber; $^{n}Bu_4NPF_6$ marked with an asterisk.*

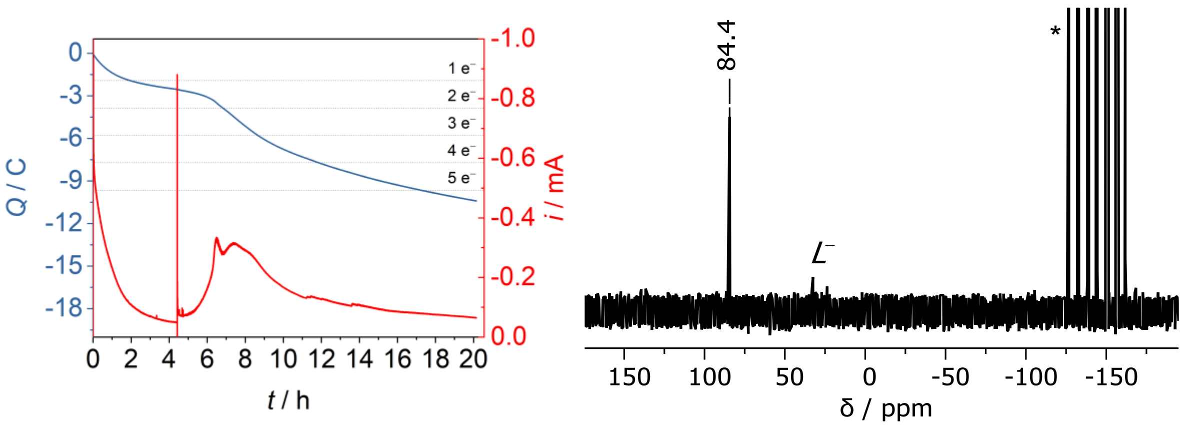

Figure S 27. *Left: Current (red) and charge (blue) over time plot of the CPE experiment of* ***1H*** *with 1 equiv. DBU in THF, $n_{\mathbf{1H}}$ = 20 µmol, I = 0.2* M *$^{n}Bu_4NPF_6$, E = −3.08 V vs $Fc^{+|0}$, N_2 atmosphere. Right: ^{31}P-NMR spectrum of the resulting solution in the working chamber; $^{n}Bu_4NPF_6$ marked with an asterisk.*

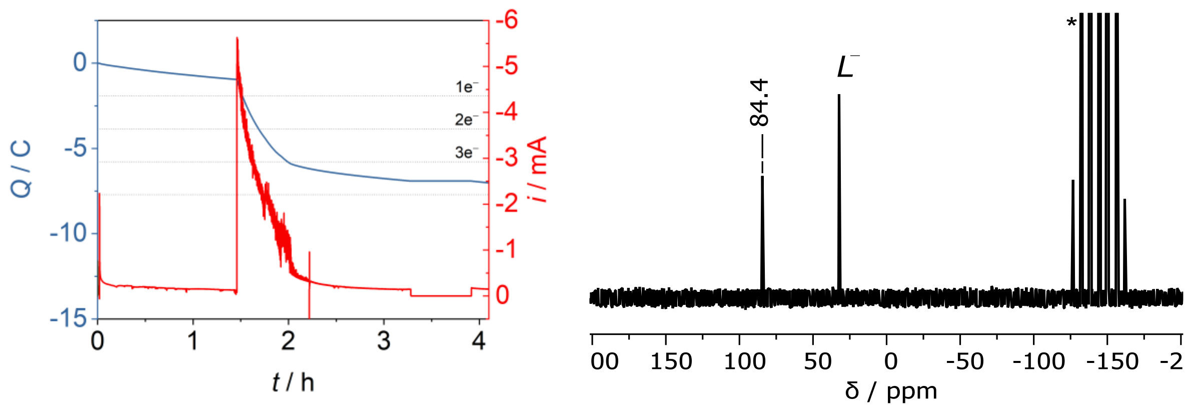

Figure S 28. *Left: Current (red) and charge (blue) over time plot of the CPE experiment of* ***1H*** *with 1 equiv. DBU in THF, n_{1H} = 20 µmol, I = 0.2 M $^{n}Bu_4NPF_6$, E = −3.15 V vs $Fc^{+|0}$, N_2 atmosphere, BDD working electrode. Right: ^{31}P-NMR spectrum of the solution in the working chamber; $^{n}Bu_4NPF_6$ marked with an asterisk.*

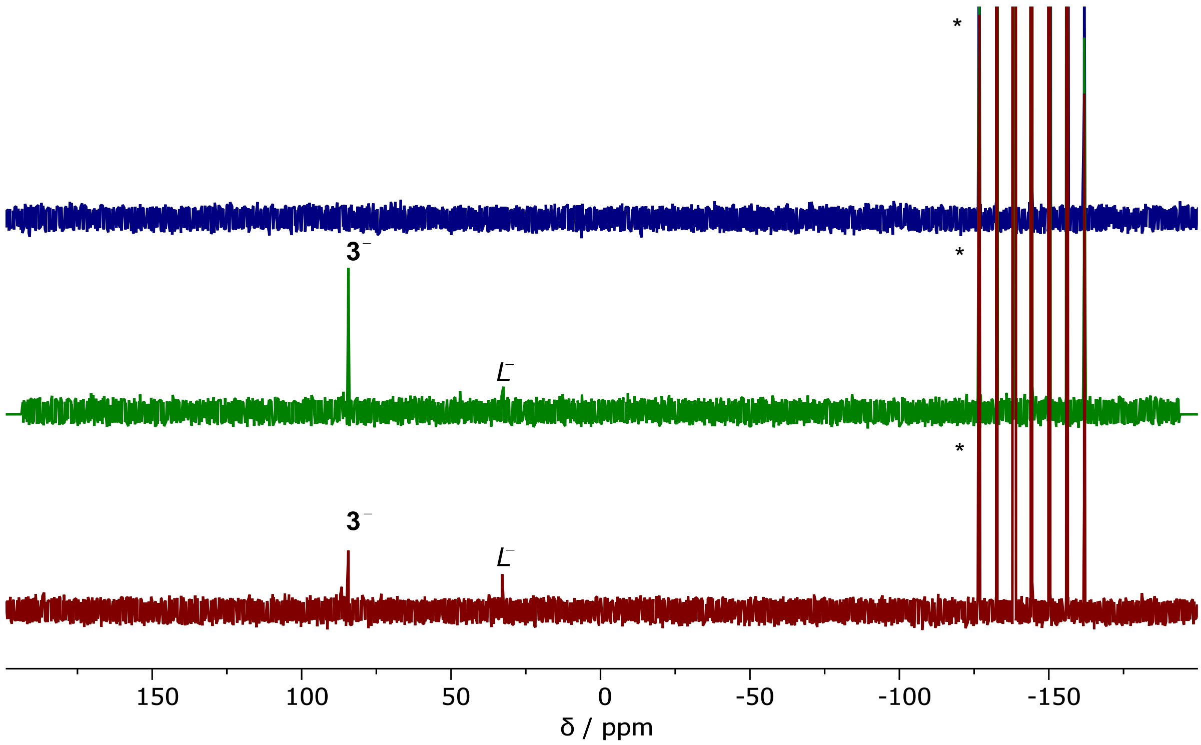

Figure S 29. *^{31}P-NMR-Spectra of the solution of* ***1H*** *with 1 equiv. DBU after injection of two electrons (blue), 3 electrons (green) and 4 electrons (red) in THF, n_{1H} = 20 µmol, I = 0.2 M $^{n}Bu_4NPF_6$, E = −2.82 V vs $Fc^{+|0}$, N_2 atmosphere; $^{n}Bu_4NPF_6$ marked with an asterisk.*

Quantification of ammonium by ^{1}H NMR spectroscopy

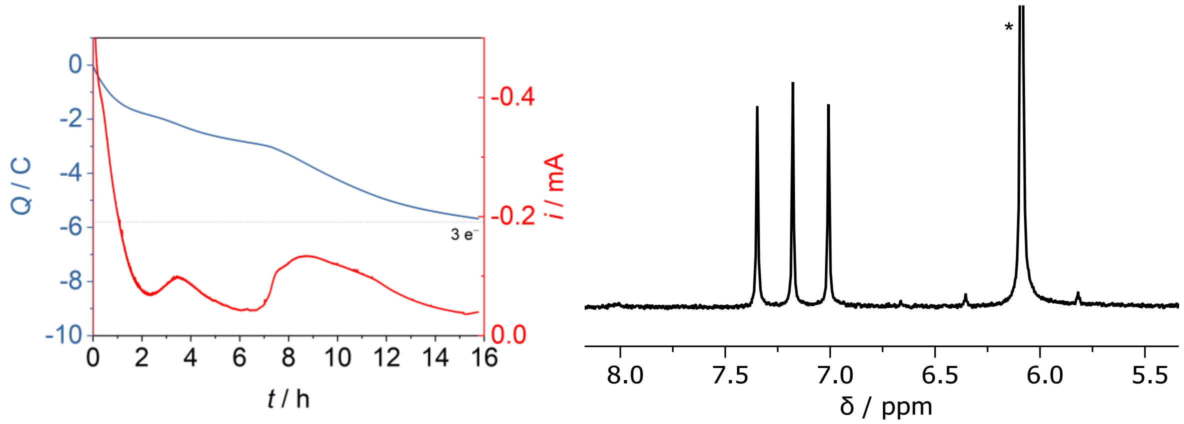

Figure S 30. *Left: Current (red) and charge (blue) over time plot of the CPE experiment of* **1H** *with 1 equiv. DBU in THF, n_{1H} = 20 µmol, I = 0.2 M $^{n}Bu_4NPF_6$, E = −2.93 V vs $Fc^{+|0}$, N_2 atmosphere. Right: ^{1}H-NMR spectrum after work-up, DMSO-d_6; TMB marked with an asterisk.*

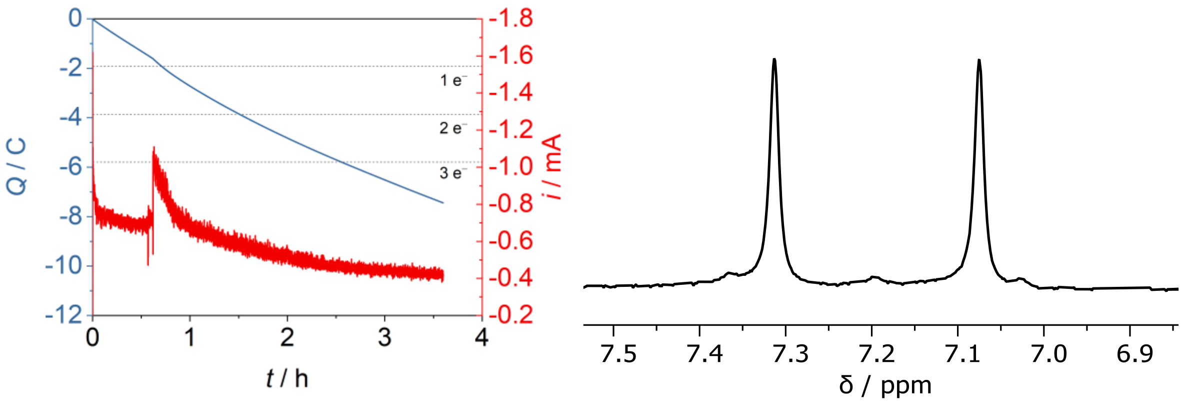

Figure S 31. *Left: Current (red) and charge (blue) over time plot of the CPE experiment of* **1H** *with 1 equiv. DBU in THF, n_{1H} = 20 µmol, I = 0.2 M $^{n}Bu_4NPF_6$, E = −2.69 V vs $Fc^{+|0}$, $^{15}N_2$ atmosphere. Right: ^{1}H-NMR spectrum after work-up, DMSO-d_6.*

IR spectra

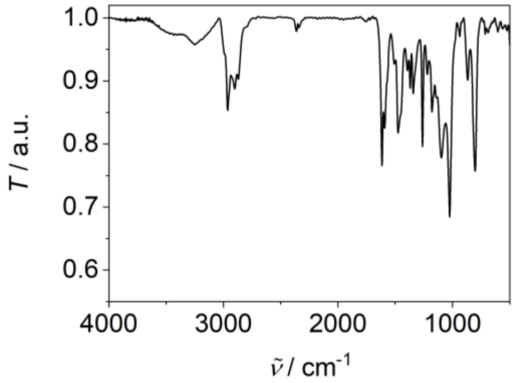

Figure S 32. *KBr IR spectrum of* **1H**.

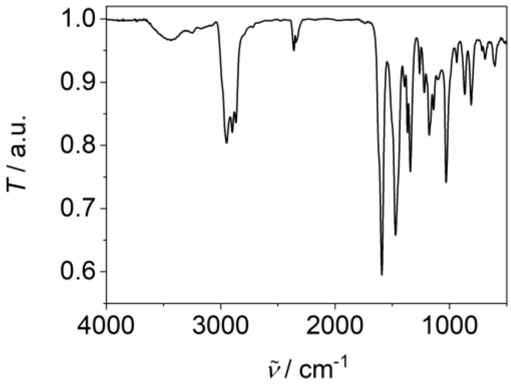

Figure S 33. *KBr IR spectrum of **2H**.*

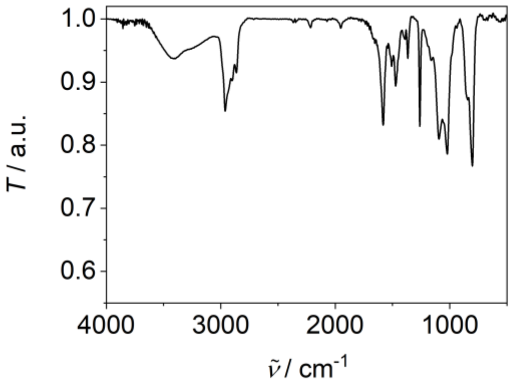

Figure S 34. *KBr IR spectrum of $\mathbf{3^K}$.*

Mass spectra

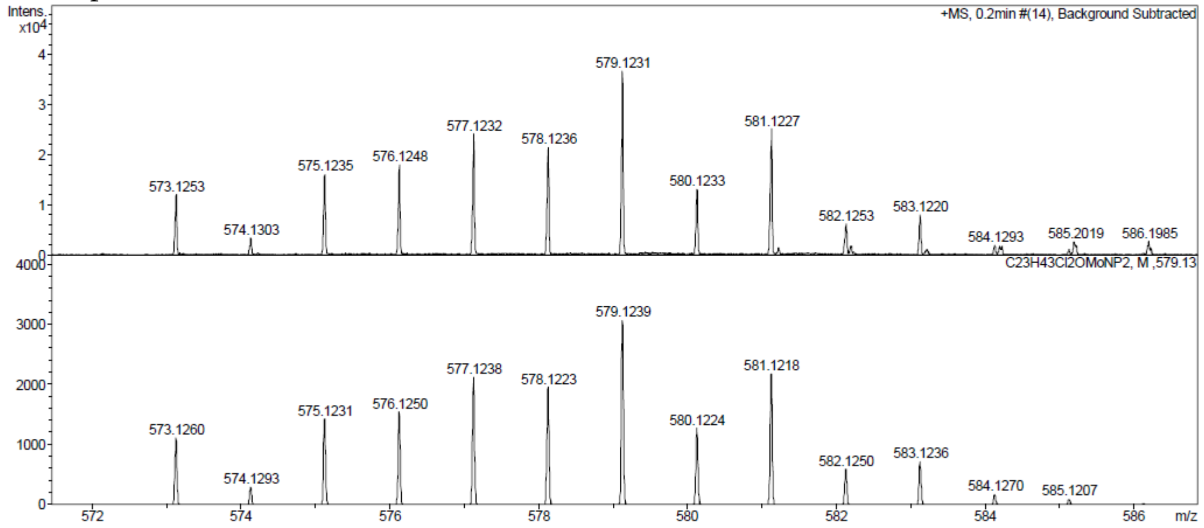

Figure S 35. *HR-ESI mass spectrum of the [M−Cl]$^+$ fragment of **1H** (top) and the corresponding simulated spectrum (bottom).*

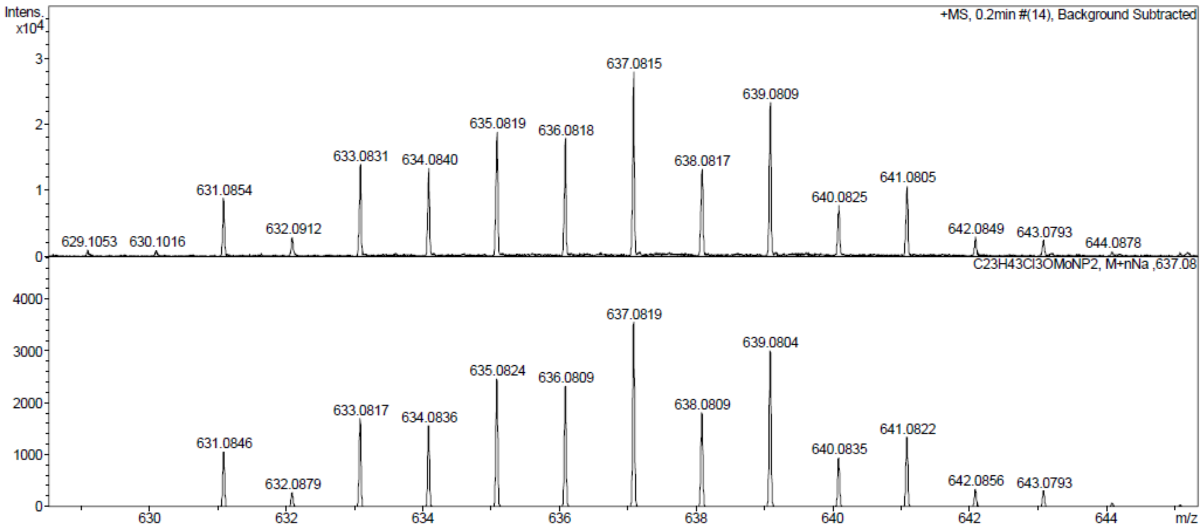

Figure S 36. *HR-ESI mass spectrum of the [M+Na]$^+$ fragment of* ***1H*** *(top) and the corresponding simulated spectrum (bottom).*

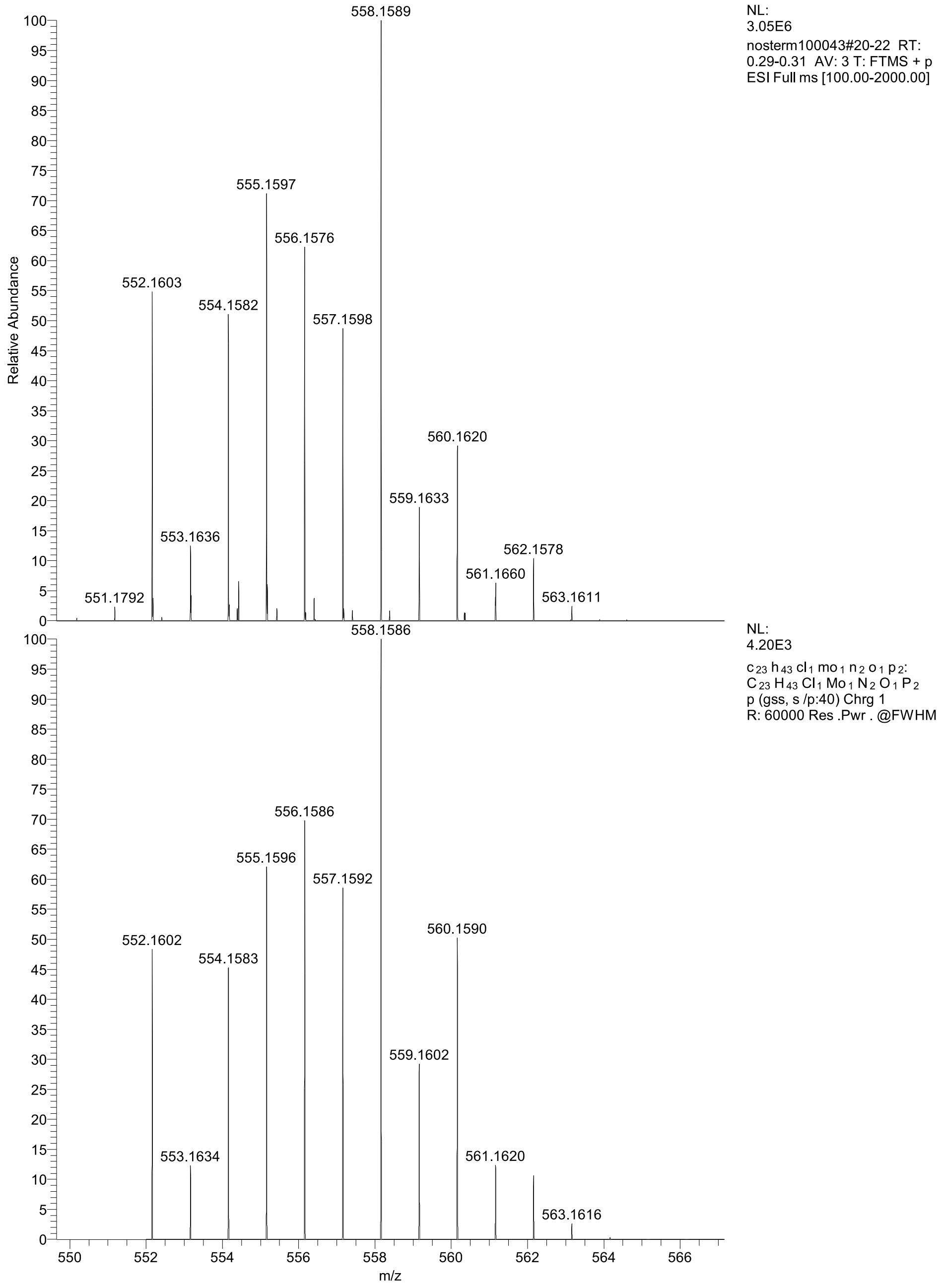

Figure S 37. *HR-ESI mass spectrum of the [M−Cl]$^+$ fragment of* ***2H*** *(top) and the corresponding simulated spectrum (bottom).*

Quantification of H_2

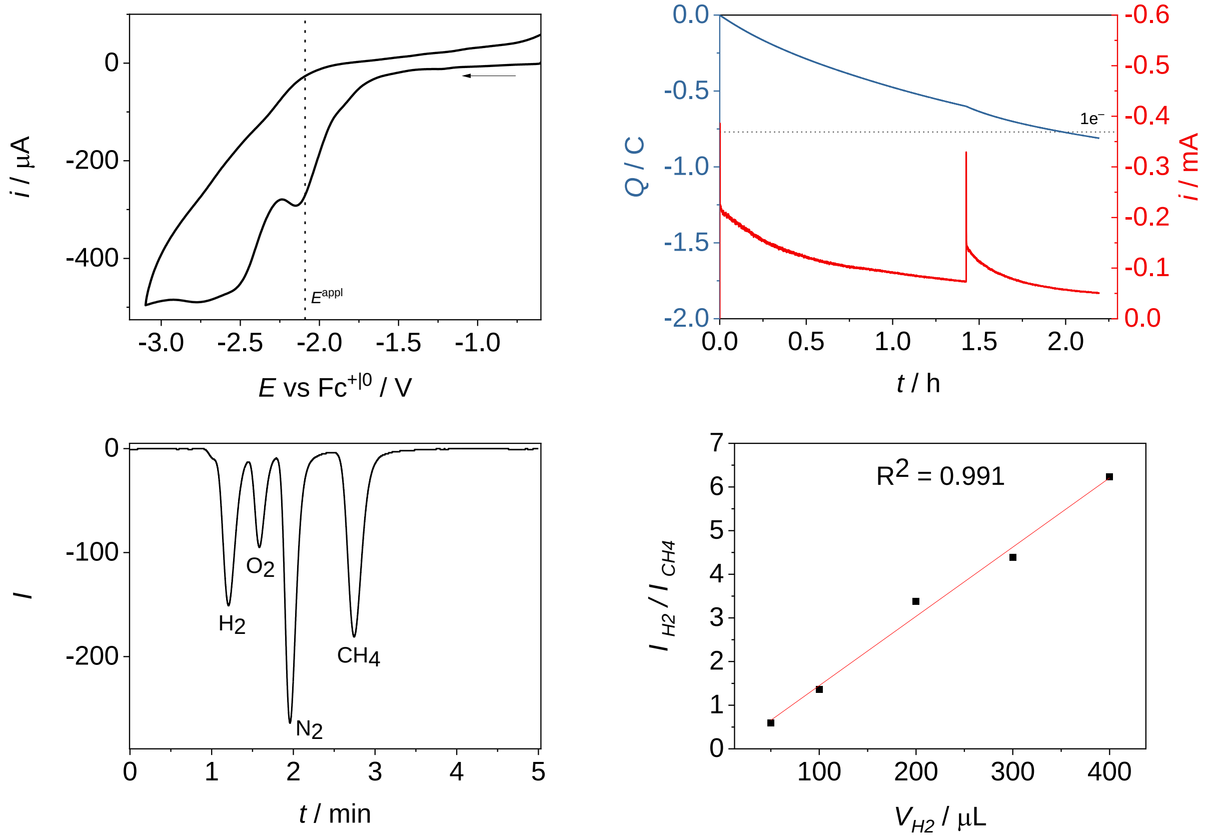

Figure S 38. *CV data of **1H** prior to the CPE experiment (top left, v = 0.1 Vs^{-1}); current (red) and charge (blue) over time plot of the CPE experiment (top right, E = −2.1 V vs $Fc^{+|0}$) of **1H** in THF; n_{1H} = 8 µmol, I = 0.2 M $^{n}Bu_4NPF_6$, Ar atmosphere. Bottom left: GC-TCD trace of the headspace after the CPE experiment. Bottom right: calibration curve to quantify the amount H_2, ratio of the H_2 peak over the methane peak (internal standard) vs the amount of H_2 injected into the cell.*

X-Ray Crystallography

X-ray data were collected with a Bruker D8 VENTURE diffractometer, equipped with a PHOTON 100 CMOS detector and two INCOATEC microfocus sources with Quazar mirror optics (Mo-K_α radiation, λ = 0.71073 Å). The structures were solved by direct methods and refined on F^2 using all reflections with SHELXL-2018/3.[5] Non-hydrogen atoms were refined anisotropically and the hydrogen atoms were placed in calculated positions and assigned to an isotropic displacement parameter of 1.2 / 1.5 U_{eq}. Absorption correction was performed by the multi-scan method with the program SADABS V2014/4.[6] The structure was uploaded to the CCDC Database and can be obtained free of charge from https://www.ccdc.cam.ac.uk, no. 2299018.

Table S 2. *Crystal data and structure refinement for* ***1H***.

empirical formula	C_{23} H_{43} Cl_3 Mo N O P_2	
formula weight	613.81	
T	100(2) K	
crystal system	Orthorhombic	
space group	$P2_12_12_1$	
Unit cell dimensions	*a* = 13.1857(7) Å	α = 90°.
	b = 14.2917(7) Å	β = 90°.
	c = 15.0821(7) Å	γ = 90°.
V	2842.2(2) $Å^3$	
Z	4	
ρ (calculated)	1.434 Mg/m^3	
F(000)	1276	
Θ-range	2.498 - 28.345°.	
hkl-range	±17, ±19, ±20	
measured reflections	70820	
unique reflections	7067 [R_{int} = 0.1178]	
data / restraints / parameters	7067 / 0 / 296	
goodness-of-fit F^2	1.035	
*R*1, *wR*2 [$I > 2\sigma(I)$]	0.0383, 0.0642	
*R*1, *wR*2 (all data)	0.0529, 0.0688	
resid. el. dens.	0.602 and−0.626 e.$Å^{-3}$	

[1] a) Evans, D. F. The Determination of the Paramagnetic Susceptibility of Substances in Solution by Nuclear Magnetic Resonance. *J. Chem. Soc.* **1959**, 2003−2005.; Bain, G. A.; Berry, J. F. Diamagnetic Corrections and Pascal's Constants. *J. Chem. Educ.* **2008**, *85*, 532−536.

[2] Krejčik, M.; Danćk, M.; Hartl, F. Simple Construction of an Infrared Optically Transparent Thin-Layer Electrochemical Cell: Applications to the Redox Reactions of Ferrocene, $Mn_2(CO)_{10}$ and $Mn(CO)_3$(3,5-di-t-butyl-catecholate)$^-$. *J. Electroanal. Chem. Interfacial Electrochem.* **1991**, *317*, 179−187.

[3] Stoll, S.; Schweiger, A. EasySpin, a Comprehensive Software Package for Spectral Simulation and Analysis in EPR. *J. Magn. Reson*. **2006**, *178*, 42−55.

[4] Mukherjee, J.; Ostermann, N.; Aniban, X.; Safianova, I.; Rotthowe, N.; Mata, R.; Siewert, I. A Nickel Complex with a Proton Responsive PNP Pincer-type Ligand as Proton-coupled Electron Transfer Reagent. *Organometallics* **2023**, *42*, 3258−3265.

[5] Sheldrick, G. M. A Short History of SHELX. Acta Crystallogr. *Acta Crystallogr., Sect. A: Found. Crystallogr.* **2008**, *64*, 112−122.

[6] SADABS V2014/4, Bruker AXS Inc., Madison, WI, USA.
